# E-Selectin-Targeted Nanomicelles via Sialic Acid Conjugation for Anti-Inflammatory Efficacy and Alleviating the Progression of Metabolic-Associated Steatotic Liver Disease

**DOI:** 10.34133/bmr.0305

**Published:** 2026-02-02

**Authors:** Congyi Zhang, Changmei Zhang, Qiong Sun, Haotian Wu, Wenze Yin, Haiyan Zhu, Shizhuan Huang, Zhihua Zhang, Yiyun Zou, Dixiang Wen, Xiaoyan Xu, Mingming Lian, Changhao Sun, Sheng Tai

**Affiliations:** ^1^ Department of Hepatic Surgery, Second Affiliated Hospital of Harbin Medical University, Harbin, China.; ^2^ Key Laboratory of Precision Nutrition and Health of Ministry of Education, School of Public Health, Harbin Medical University, Harbin, China.; ^3^ Department of Pharmaceutics, Harbin Medical University-Daqing Campus, Daqing, China.; ^4^ Department of Children’s and Adolescent Health, Public Health College, Harbin Medical University, Harbin, China.

## Abstract

**Background:** Metabolic-associated steatotic liver disease (MASLD), including metabolic dysfunction-associated steatohepatitis (MASH), is a growing health concern characterized by liver inflammation, fibrosis, and endothelial dysfunction. Targeted therapies are essential to address these issues and improve treatment outcomes. **Methods:** A sialic acid (SA)-modified nanomicelle system (SA-PEG-ALA) was developed to target liver sinusoidal endothelial cells (LSECs) via the E-selectin (SELE). Molecular docking and surface plasmon resonance (SPR) were used to confirm the binding interaction between SA and SELE. In vitro assays using LSECs and steatotic hepatocytes were conducted to evaluate the cellular uptake and therapeutic efficacy of SA-PEG-ALA. In vivo studies using an HFHC-induced MASH mouse model were carried out to evaluate the distribution and therapeutic outcomes of SA-PEG-ALA. Additionally, RNA sequencing was performed to explore the molecular mechanisms underlying its effects. **Results:** Molecular docking and SPR analyses confirmed that SA effectively binds to SELE, facilitating the targeted delivery of ALA to LSECs. In vitro, SA-PEG-ALA showed substantially higher uptake in LSECs compared to other formulations. In vivo, SA-PEG-ALA demonstrated superior targeting of the liver and showed enhanced therapeutic effects compared to PEG-ALA, significantly alleviating steatosis, liver inflammation, and fibrosis in the MASH model. Mechanistically, SA-PEG-ALA interacted with HSP70, enhancing its stability and promoting the binding of HSP70 to IκBα, which contributed to inhibition of NF-κB signaling pathway. **Conclusion:** SA-PEG-ALA offers a promising targeted therapeutic strategy for MASLD, with improved liver targeting, anti-inflammatory, and antifibrotic effects, highlighting its potential for treating MASLD.

## Introduction

Metabolic-associated steatotic liver disease (MASLD) is a growing global health issue, encompassing a spectrum of liver conditions ranging from simple hepatic steatosis to more severe stages such as metabolic dysfunction-associated steatohepatitis (MASH), fibrosis, cirrhosis, and ultimately hepatocellular carcinoma (HCC) [1–3]. It is estimated that MASLD affects 30% of the global population, with this incidence expected to rise substantially in the coming years [4]. Moreover, the increasing prevalence of obesity, diabetes, and metabolic syndrome has further amplified the burden of MASLD, making it a leading cause of liver-related morbidity and mortality [5,6]. Although lifestyle modifications targeting obesity and metabolic risk factors are commonly employed, therapeutic options specifically targeting the pathophysiological mechanisms driving MASLD remain scarce.

Endothelial cell dysfunction is a critical pathological feature of MASLD and plays a central role in disease progression [7,8]. When endothelial cells are activated due to metabolic disturbances, they express adhesion molecules like selectins, which mediate immune cell adhesion and recruitment. The selectin family comprises 3 key cell adhesion molecules: L-selectin (SELL), predominantly expressed by leukocytes, and E-selectin (SELE) and P-selectin (SELP), which are up-regulated on activated endothelial cells [9–11]. These selectins mediate interactions with glycosylated ligands. This immune cell infiltration aggravates liver inflammation, exacerbating the progression of steatosis to more severe stages like MASH and fibrosis. Targeting the dysfunctional endothelial cells and modulating the immune response through SELE inhibition holds great promise for slowing or even halting the progression of MASLD.

α-Lipoic acid (ALA), a potent antioxidant and anti-inflammatory agent, has been investigated for its ability to alleviate oxidative stress and mitigate endothelial dysfunction in cardiovascular diseases [12]. Previous study showed that ALA could activate ALDH2-dependent Nrf1-FUNDC1 signaling, which is crucial for maintaining mitochondrial function and reducing oxidative stress in endothelial cells [13]. Furthermore, ALA could reduce chemotherapy-induced side effects and chemoresistance, making it a promising adjunct in cancer therapy [14]. Despite these benefits, the pharmacological efficacy of ALA in MASLD is limited by its low water solubility, poor bioavailability, and lack of targeting specificity, which prevents it from effectively addressing the endothelial dysfunction and immune activation associated with MASLD.

To address these limitations, we have designed a novel approach by incorporating sialic acid (SA) as a targeted ligand to enhance the specificity and efficacy of ALA. SA consists of a 9-carbon backbone and a negatively charged terminal group structurally, and is found as the termini of mammalian cell surface glycoproteins and glycolipids [15,16]. Notably, SA is a natural ligand for selectin, facilitating the binding of leukocytes to the endothelial cells. Recent study has shown that the interaction between SA and selectins promoted the adhesion of tumor cells to endothelial cells, a critical step in the metastatic spread of cancer [17]. Hence, we hypothesize that SA has the potential to specifically bind to LSECs expressing selectins in the context of MASLD.

**Fig. 1.**
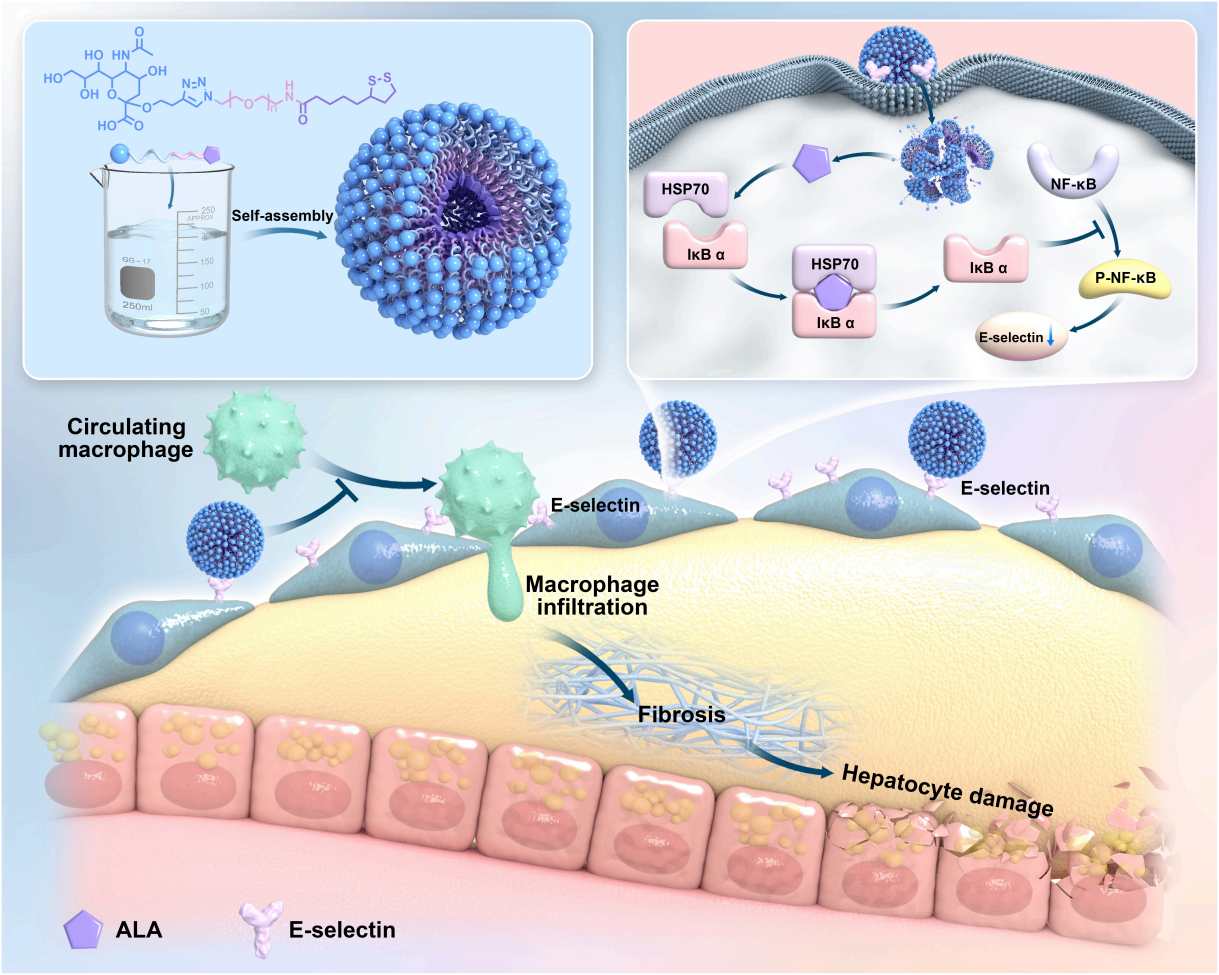
A PEG-based nanocarrier system that incorporated SELE targeting functionality was synthesized and designed and offered hepatoprotective effects in MASLD.

In this study, we developed and evaluated SA-PEG-ALA, a targeted nanomicelle drug delivery system for the treatment of MASH. We hypothesized that SA-PEG-ALA exhibited significant therapeutic potential by enhancing the liver-specific delivery of ALA, with a focus on targeting LSECs via SELE. Through its targeted action, SA-PEG-ALA not only alleviated steatosis, inflammation, and liver injury but also mitigated fibrosis progression in MASH. By leveraging SELE as a target, SA-PEG-ALA offered a more precise therapeutic approach and afforded a novel remedy in the treatment of MASLD (Fig. 1).

## Materials and Methods

### Human liver samples

Liver samples were randomly obtained from individuals undergoing bariatric surgery in the Second Affiliated Hospital of Harbin Medical University. These samples were then processed with hematoxylin and eosin (H&E) staining and independently examined by 2 pathologists using standard histological criteria to ensure unbiased selection. All procedures involving the collection of human samples were approved by the Ethics Committee of Harbin Medical University (HMUIRB2024035). Written informed consent was obtained from the participants or their family members in accordance with the principles outlined in the Declaration of Helsinki. Detailed clinicopathological features of liver samples involved in this study were shown in Table S1.

### Animal models and animal experiment management

Male C57BL/6 mice, aged 4 weeks, were purchased from Vital River (Beijing, China) and adaptively bred for 2 weeks. MASH model was established by feeding a high-fat, high-cholesterol (HFHC) diet (42% fat, 14% protein, 44% carbohydrate, and 2% cholesterol; IMA2019001, Nantong, China) for 20 weeks. To establish another MASH model, the mice received a methacholine-deficient (MCD) diet (TP3005G, Nantong, China) diet for 4 weeks. To evaluate the therapeutics effects of SA-PEG-ALA on HFHC-induced MASH mice, the animals were administered with saline, SA-PEG, free ALA, PEG-ALA, and SA-PEG-ALA (free ALA dose: 0.5 mg/kg; SA-PEG dose: 2.2 mg/kg; PEG-ALA dose: 2.0 mg/kg; SA-PEG-ALA dose: 2.7 mg/kg) on every other day over a period of 3 weeks. All mice were kept in the specific pathogen-free (SPF) animal room with a 12-h light/dark cycle, maintained at a temperature range of 18 to 22 °C and humidity levels of 50% to 60%. The animal experiment was approved by the Animal Ethics Committee of Harbin Medical University (HMUIRB2024035).

### RNA-sequencing analysis

RNA sequencing (RNA-seq) was performed on whole liver from both chow- and HFHC diet-fed mice and LSECs treated with control, cocultured with steatotic 7702 cells, and cocultured with steatotic 7702 cells + SA-PEG-ALA. Total RNA was extracted using TRIzol reagent (catalog no. 15596018CN, Invitrogen, USA) according to the manufacturer’s instructions. After that, library preparation was performed following the standard Illumina instruction (VAHTS Universal V6 RNA-seq Library Prep Kit for Illumina). Agilent 4200 bioanalyzer was employed to evaluate the concentration and size distribution of cDNA library before sequencing with an Illumina NovaSeq 6000. The protocol of high-throughput sequencing was fully according to the manufacturer’s instructions (Illumina). The raw reads were filtered by Seqtk before mapping to genome using Hisat2 (version:2.0.4). The fragments of genes were counted using stringtie (v1.3.3b) followed by TMM (trimmed mean of M values) normalization. Significant differential expressed genes (DEGs) were identified as those with a false discovery rate (FDR) value above the threshold (*Q* < 0.05) and fold change > 1.5 using R software.

### H&E staining

Consecutive 5-μm paraffin sections from both normal and MASH human tissues were deparaffinized through a graded ethanol series (100%, 95%, 75%), stained with hematoxylin, and subsequently dehydrated via an ascending ethanol gradient (75% to absolute) prior to clearing in xylene and mounting with neutral balsam for microscopic observation.

### SELE and ICAM-1 expression

Subsequently, total protein was extracted from the tissues for subsequent analysis. Equal amounts of proteins (40 μg per well) and protein marker were taken for sodium dodecyl sulfate–polyacrylamide gel electrophoresis (SDS-PAGE) electrophoresis and then transferred to a polyvinylidene difluoride (PVDF) membrane. The membrane was blocked with 5% bovine serum albumin (BSA) at room temperature for 1 h and then incubated overnight on a shaker at 4 °C with SELE, ICAM-1 (1:1,000). After thorough washing with TBST, the membrane was incubated with secondary antibody (1:50,000) at room temperature in the dark for 1 h. The enhanced chemiluminescence (ECL) method was used for color development. The ImageJ software was used to analyze the gray value of the target band, and β-tubulin was used as an internal reference for standardized quantification.

### SELE expression of Western blot

Liver tissues and cells were efficiently lysed with radioimmunoprecipitation assay (RIPA) lysis buffer (89900, Thermo Fisher Scientific), supplemented with protease inhibitor cocktail (78445,Thermo Fisher Scientific). Total protein was extracted from the supernatant by centrifuging at 10,000*g* for 30 min at 4 °C, and the BCA Protein Assay Kit (23227, Thermo Fisher Scientific) was used to measure the concentration of protein. Protein separation was carried out using a 10% SDS-PAGE. Then, the proteins were transferred to PVDF membranes, which were blocked with 5% BSA in tris-buffered saline with Tween (TBST). The membranes were incubated with the primary antibody (SELE, Abcam) targeting the protein of interest overnight at 4 °C, followed by incubation with the relevant horseradish peroxidase (HRP)-conjugated secondary antibody for 1 h at 37 °C. Finally, the bands were detected using the ECL kit (32209, Thermo Fisher Scientific).

### Immunohistochemical staining and immunofluorescence assay

Liver tissues and cells were fixed in 10% formalin, dehydrated, embedded in paraffin, and sectioned into 5-μm slices. The sections were processed for dewaxing, rehydration, antigen retrieval, and blocking. Following these steps, the sections were incubated with primary antibodies overnight at 4 °C. On the following day, for immunohistochemistry (IHC), the sections were incubated with secondary antibodies for 1 h. Subsequently, the sections were stained with diaminobenzidine (DAB kit; Vector Laboratories, Burlingame, CA, USA) and counterstained with hematoxylin (Sigma, St. Louis, MO, USA), according to the manufacturer’s instructions. The staining intensity was graded as 0 (negative), 1 (weak), 2 (moderate), or 3 (strong). The percentage scores were defined as follows: 0 for <5%, 1 for 5% to 25%, 2 for 26% to 50%, 3 for 51% to 75%, and 4 for >75%. The histological score for each sections was calculated using the following formula:

Historical score = proportion score×intensity score.

For tissue immunofluorescence (IF), the sections were incubated with fluorescent secondary antibodies for 1 h. Afterward, the nuclei were stained with 4′,6-diamidino-2-phenylindole (DAPI). ImageJ software was used to quantify the integrated fluorescence intensity of the labeled antigens in the acquired images. The mean gray value was calculated using the following formula:

Mean gray value = Integrated density/Area.

For cellular IF staining analysis, cells were fixed with 4% paraformaldehyde (PFA), permeabilized with 0.25% Triton for 20 min to enhance membrane permeability, and then blocked with 10% BSA at 37 °C for 1 h. The primary antibody was applied and incubated overnight at 4 °C. The corresponding fluorescent secondary antibody was then incubated at 37 °C for 1 h. After that, the nuclei were stained with DAPI. Finally, images were captured using a confocal laser scanning microscope (TCSSP8; Leica). Information of all the primary antibodies used in this study is provided in Table S2.

### Cell lines and cell culture

Human hepatic cell line HL-7702 was obtained from the Chinese Academy of Science (Shanghai, China). Human liver sinusoidal endothelial cells (LSECs) were purchased from the Zhong Qiao Xin Zhou Biotechnology (PRI-H-00130, Shanghai, China). THP-1 cells (Procell Life Science & Technology Co. Ltd., China) were cultured in RPMI 1640 medium (Invitrogen, USA) containing 10% fetal bovine serum (FBS) at 37 °C. HL-7702 cells were maintained in Dulbecco’s modified Eagle’s medium (Gibco, USA), and LSECs were maintained in Endothelial Cell Medium (M200500, Thermo Fisher Scientific). Both were cultured at 37 °C in a 5% CO_2_ atmosphere, supplemented with 10% FBS (Gibco, USA) and 1% antibiotics (100 U/ml penicillin and 100 μg/ml streptomycin).

### Oil Red O staining

600 μM oleic acid (Sigma-Aldrich) treated HL-7702 cells for 36 h. Then, gently discard the culture medium and wash with PBS for 3 times. After that, aspirate the PBS and add 10% PFA for fixation for 30 min at room temperature. Add an appropriate amount of staining wash solution to cover the cells for 20 s. Then, remove the staining wash solution and add a sufficient amount of Oil Red O staining working solution, staining for 10 to 20 min. Afterward, remove the Oil Red O staining working solution, add an appropriate amount of staining wash solution, and let it sit for 30 s. Then, remove the wash solution and wash with PBS for 20 s. We added an appropriate amount of PBS to evenly cover the cells and then observed and photographed them under a microscope. In addition, the expression of SELE protein was detected by IF and Western blot (WB).

### Adhesion of monocytes to LSECs

LSECs were cocultured with steatotic 7702 cells for 24 h. The medium was replaced with 3,3′-dioctadecyloxacarbocyanine perchlorate (DiO)-labeled resting THP-1 cells (1 × 10^5^/ml) for 2 h at gentle shaking of 50 rpm. After that, LSECs were washed with PBS and fixed in 4% PFA. The adherent DiO-labeled monocytes were imaged by confocal microscopy (OLYMPUS, FLUOVIEW, FV3000, Japan). The images were quantified using the ImageJ software.

### Flow cytometric analysis of apoptosis

The FITC Annexin V Apoptosis Detection Kit (88-8005-74, Thermo Fisher Scientific) was used to detect cell apoptosis. The LSECs were collected after incubation with steatotic 7702 cells. The cells were then resuspended in 400 μl of binding buffer, followed by staining with 5 μl of Annexin V-FITC (fluorescein isothiocyanate) solution and 3 μl of propidium iodide (PI) for 15 min in the dark at room temperature. A flow cytometer (Beckman) was used to analyze the positive cells.

### Synthesis of SA-PEG-ALA

Lipoic acid (1 g, 5 mmol), N-hydroxysuccinimide (575 mg, 5 mmol), and 1-(3-dimethylaminopropyl)-3-ethylcarbodiimide hydrochloride (960 mg, 5 mmol) were dissolved in CH_2_Cl_2_ (20 ml) under an argon atmosphere in a round-bottomed flask. The mixture was stirred at room temperature for 30 min. Compound 1 (2.3 g, 3.8 mmol) was added and stirred at room temperature overnight. The resulting crude mixture was washed with 1 M aqueous HCl. The organic layer was dried over sodium sulfate and filtered, and the excess solvent was removed under reduced pressure. The crude product was then purified by flash column chromatography on silica gel using a MeOH/CH_2_Cl_2_ solvent mixture (1:10), yielding the pure product 2. Compound 2 (500 mg, 0.62 mmol) was dissolved in tert-butanol (10 ml) in a round-bottomed flask. A solution of compound 3 (671 mg, 1.86 mmol) in H_2_O (10 ml), along with 0.05 mol aqueous CuSO_4_ (380 μl, 0.19 mmol) and 0.05 mol aqueous sodium ascorbate (1.24 ml, 0.62 mmol), was added to the flask. The mixture was stirred at room temperature overnight. Afterward, the solvent from the resulting crude mixture was removed with vacuum, the residue was dissolved in CH_2_Cl_2_, and insoluble impurities were filtered out. The crude product was purified by flash column chromatography using silica gel with a solvent mixture of MeOH/CH_2_Cl_2_ (1:100 to 1:2), yielding the pure product SA-PEG-ALA.

### Synthesis of PEG-ALA

Lipoic acid (500 mg, 2.5 mmol), N-hydroxysuccinimide (288 mg, 2.5 mmol), 1-(3-dimethylaminopropyl)-3-ethylcarbodiimide (480 mg, 2.5 mmol), and dichloromethane (20 ml) were successively added into a 100-ml single-necked flask. Under the protection of argon, the reaction was carried out at room temperature for 30 min. Then, amino-polyethylene glycol monomethyl ether 1200 (2.3 g, 1.9 mmol) was added, and the reaction was allowed to proceed at room temperature overnight. After the reaction was completed, dichloromethane was added for dilution. The mixture was washed 3 times with a saturated aqueous sodium chloride solution and then passed through a silica gel column (first eluted with ethyl acetate and then with dichloromethane/methanol = 10/1). As a result, 1.7 g of a yellow oily compound, PEG-ALA, was obtained, with a yield of 64.4%.

### Synthesis of SA-PEG

Azide acetic acid (121.2 mg, 1.2 mmol), 4-dimethylaminopyridine (DMAP; 183 mg, 1.5 mmol), and 1-(3-dimethylaminopropyl)-3-ethylcarbodiimide hydrochloride (288 mg, 1.5 mmol) were dissolved in CH_2_Cl_2_ (20 ml) under an argon atmosphere in a round-bottomed flask. The mixture was stirred at room temperature overnight. The resulting crude mixture was washed with saturated sodium chloride. The organic layer was dried over anhydrous sodium sulfate, filtered, and concentrated under reduced pressure to remove the solvent. The crude product was purified by flash column chromatography on silica gel (MeOH/CH_2_Cl_2_ 1:10) to afford the pure product compound 1. After that, compound 1 (140 mg, 0.18 mmol) was dissolved in tert-butanol (10 ml) in a round-bottomed flask. Alkynyl-SA (130 mg, 0.36 mmol) in H_2_O (10 ml), 0.05 M aqueous CuSO_4_ (100 μl, 0.05 mmol), and sodium ascorbate (36 mg, 0.18 mmol) were added. The mixture was stirred at 50 °C overnight. The crude product was purified by flash column chromatography on silica gel (MeOH/CH_2_Cl_2_ 1:10 to 1:5) to afford the pure product compound 2 (SA-PEG).

### Preparation, characterization, and release profile of nanomicelles

SA-PEG-ALA and PEG-ALA compounds were precisely weighed and introduced into ultrapure water. Subsequently, the mixture underwent sonication to ensure complete dissolution, resulting in the preparation of 1 mg/ml SA-PEG-ALA and PEG-ALA nanomicelles. Transmission electron microscope (TEM) (JEM1200EX, Japan) was used to observe the morphology of the nanomicelles. At room temperature, a Malvern nanoparticle size analyzer (Malvern, UK) was used to measure the particle size, zeta potential, and polydispersity index (PDI) of the nanomicelles. The morphological changes of the nanomicelles were observed within 30 d to evaluate their stability. Finally, an ultraviolet–visible (UV–Vis) spectrophotometer was used for full-wavelength ultraviolet scanning.

The release profile of ALA from SA-PEG-ALA or PEG-ALA nanomicelles was investigated using a dialysis method. Briefly, 1 ml of the nanomicellar formulation was loaded into a dialysis tube (molecular weight cutoff: 10 kDa) and dialyzed against 200 ml of PBS (pH 7.4) at 37 °C under continuous shaking at 50 rpm. At predetermined time intervals, 1 ml of the external release medium was withdrawn and replaced with an equal volume of fresh prewarmed PBS. The concentration of released ALA in the withdrawn samples was directly quantified by using a UV–Vis spectrophotometer.

The analysis of ALA concentration was performed using high-performance liquid chromatography (HPLC). The chromatographic conditions were as follows: A mixture of 0.005 M potassium dihydrogen phosphate solution (pH adjusted to 3.1 with phosphoric acid), methanol, and acetonitrile (25:37.5:37.5, v/v/v) served as the mobile phase under isocratic elution. The flow rate was set at 1.0 ml/min, the detection wavelength was 215 nm, the column temperature was maintained at 30 °C, and the injection volume was 10 μl. ALA standard was dissolved and diluted with the mobile phase to prepare a stock solution with a concentration of approximately 1,000 μg/ml, which was subsequently diluted to generate a series of standard working solutions at concentrations of 5, 10, 50, 100, and 200 μg/ml. All solutions were filtered through a 0.45-μm membrane filter prior to analysis. After system equilibration, the standard working solutions were injected in ascending order of concentration. The retention time and peak area of α-lipoic acid were recorded. A standard curve was constructed by plotting the concentration of the standard solutions (X, μg/ml) against the corresponding peak area (Y), followed by linear regression. LSECs cultured in 6-well plates were pretreated with or without varying concentrations of free SA for 12 h, followed by incubation with 0.5 mg/ml SA-PEG-ALA or PEG-ALA for an additional 12 h. After that, the cells were washed with PBS, harvested, and homogenized. The homogenates were centrifuged at 12,000 rpm for 10 min, and the resulting supernatants were collected for the determination of ALA content by HPLC.

### Molecular docking

Molecular docking studies were performed to investigate the interactions between SA and SELE, as well as between ALA and HSP70. The procedures were carried out using AutoDockTools 1.5.2. The 3-dimensional structure of SELE was obtained from the AlphaFold database. The crystal structure of human HSP70 was retrieved from the Protein Data Bank (PDB ID: 5BN8). Both protein structures were prepared for docking by adding polar hydrogen atoms and assigning Gasteiger charges. The 3-dimensional structure of SA was constructed using ChemDraw 20.0 and subsequently energy-minimized. The structure of ALA was prepared similarly. All rotatable bonds in the ligands were defined as flexible during the docking simulation. Docking grids were defined to encompass the putative binding sites of the respective proteins. For SELE, the grid box was centered at *x* = 0, *y* = 0, *z* = 0 with dimensions of 126 × 126 × 126 grid points and a spacing of 0.375 Å. For HSP70, the grid box was centered at *x* = 14.1, *y* = −13.0, *z* = 27.5 with identical dimensions. The docking calculations were performed using the Lamarckian Genetic Algorithm. The resulting binding poses were clustered and ranked according to their calculated binding affinity (Δ*G*, kcal/mol). The pose with the most favorable binding energy from the most populous cluster was selected for further analysis. Molecular visualizations and rendering of the docking complexes were conducted using PyMOL 2.0.

### Surface plasmon resonance

The activator is prepared by mixing 400 mM 1-ethyl-(3-dimethylaminopropyl) carbodiimide (EDC) and 100 mM N-hydroxysuccinimide (NHS) immediately prior to injection. The chip is activated for 240 s with the mixture at a flow rate of 20 μl/min. Dilute SELE protein to 50 μg/ml in immobilization buffer, then injected to sample channel at a flow rate of 20 μl/min. The chip is deactivated by 1 M ethanolamine hydrochloride at a flow rate of 20 μl/min for 240 s. A series of SA-SS-ALA solutions (100, 50, 25, 12.5, 6.25, 3.125, 1.563, and 0.781 μM) was prepared in the same analyte buffer by serial dilution. SA-SS-ALA is injected to a sample channel at a flow rate of 20 μl/min for an association phase of 240 s, followed by 360-s dissociation. The association and dissociation process are all handling in the analyte buffer. Eight cycles of analyte are repeated according to analyte concentrations in ascending order. After each cycle of interaction analysis, the sensor chip surface should be regenerated completely with 10 mM glycine-HCl as injection buffer at a flow rate of 150 μl/min for 10 s to remove the analyte. Thereafter, the next concentration cycle of the analyte SA-SS-ALA was initiated by repeating the injection and regeneration steps.

### Ultrathin transmission

LSECs were seeded in culture plates and treated with 0.4 mg/ml SA-PEG-ALA for 24 h. After treatment, the culture medium was removed, and the cells were rinsed 3 times with PBS. Glutaraldehyde fixative was then slowly added along the edge of the culture flask until the cells were just covered, and the cells were fixed at 4 °C for 30 min. Afterward, the cells were gently scraped into the glutaraldehyde solution. The cell suspension was centrifuged at 2,500*g* for 3 to 5 min, and the supernatant was discarded. Next, 1 ml of 2.5% glutaraldehyde fixative was slowly added along the wall of the tube, and the cells were fixed at 4 °C overnight. Finally, ultrathin sections were prepared using a Leica UC7 ultramicrotome, and images were captured with a JEM-1200EX TEM.

### Cellular uptake of confocal imaging

When the LSECs in a T25 flask reach confluence, one-sixth or one-fifth of the cells were transferred into a 12-well plate, with cell culture inserts placed in advance. After 24 h, the culture medium was removed, and 20 μl of SA-PEG-ALA@DiO, PEG-ALA@DiO, and SA-PEG@DiO (10 mg/ml) was added to each well. After 4 h of incubation, the 12-well plate was removed, and the culture medium was discarded. PFA (10%) was added for fixation for 30 min at room temperature. The cells were then washed 3 times with PBS, each wash lasting 5 min. The cell culture inserts were carefully removed, and 2.5 μl of DAPI was added to each insert. The inserts were then mounted on slides, and images were captured using a confocal microscope (OLYMPUS, FLUOVIEW, FV3000, Japan).

### Flow cytometry analysis of absorption

Once the LSECs in a T25 flask reach confluence, one-third of the cells were transferred onto cell slides for 24 h. Then, 20 μl of SA-PEG-ALA@DiO, PEG-ALA@DiO, and SA-PEG@DiO (10 mg/ml) was added to each well. After 4 h, the cells were rinsed once with PBS. Next, 200 μl of 0.25% trypsin was added for 2 min of digestion, followed by the addition of 400 μl of complete culture medium to stop the digestion. The cells were then transferred to a sterile 1.5-ml Eppendorf tube and centrifuged at 1,000 rpm for 5 min. The supernatant was discarded, and 400 μl of PBS was added to resuspend the cells. Finally, the samples were analyzed using a flow cytometer (Beckman) with the FITC channel.

### Hemolysis assay

Blood was collected from the abdominal aorta of mice and placed into sterile heparinized tubes. After centrifugation, the pellet was washed several times with normal saline. The resulting pellet was then diluted to a 2% red blood cell suspension. Nanomicelles at varying concentrations were gently mixed with the 2% red blood cell suspension and incubated at 37 °C for 1 h. Following incubation, the mixture was centrifuged at 2,500 rpm for 10 min. Next, 100 μl of the supernatant from each group was transferred to a 96-well plate, and absorbance was measured at 540 nm using a microplate reader. Distilled water served as a complete positive control, while normal saline was used as a negative control. The hemolysis rate (%) = [(*A*_sample_ − *A*_negative control_)/(*A*_positive_ − *A*_negative control_)] × 100%.

### In vivo and ex vivo imaging

1,1′-Dioctadecyl-3,3,3′,3′-tetramethylindotricarbocyanine iodide (DiR) and the SA-PEG-ALA compound were accurately weighed and individually dissolved in chloroform to obtain 1 mg/ml stock solutions. The SA-PEG-ALA and DiR solutions were mixed at a volume ratio of 10:1, vortexed thoroughly, and evaporated under appropriate conditions to remove chloroform. The resulting film was hydrated with 1 ml of physiological saline, followed by ultrasonication and centrifugation at 2,000 rpm for 5 min. The supernatant was collected to obtain the purified SA-PEG-ALA@DiR nanomicelle solution. Following the intravenous injection of SA-PEG-ALA@DiR nanomicelles (10 mg/kg) via the tail vein, normal and HFHC diet-fed mice were anesthetized and sacrificed at predetermined time points (30 min, 6 h, 12 h, 24 h, and 48 h). The liver, spleen, lungs, and kidneys were then collected and subjected to fluorescence imaging using a small animal in vivo imaging system with excitation and emission wavelengths of 720 and 790 nm, respectively.

### Construction of MASH model

The MASH model was established by feeding mice an HFHC diet for 20 weeks, after which the mice received tail vein injections of SA-PEG, PEG-ALA, or SA-PEG-ALA (10 mg/ml, 20 mg/kg) every other day for 4 weeks starting at week 26. Following the treatment period, the mice were sacrificed and liver tissues were harvested for histological and protein analysis, including H&E staining, IHC examination of CD68 and α-smooth muscle actin (αSMA) expression, and WB assessment of phosphorylated and total NF-κB levels.

### Real-time fluorescence quantitative PCR

Total tissue and cell RNA was extracted using TRIzol reagent (15596026CN, Thermo Fisher Scientific), and cDNA was synthesized using High Capacity RT Kit (4374966, Thermo Fisher Scientific). Quantitative polymerase chain reaction (qPCR) was conducted using SYBR Green on a 7500 Fast PCR System, and the mRNA expression levels were normalized to glyceraldehyde-3-phosphate dehydrogenase (GAPDH). The primer information was listed in Table S3.

### Enzyme-linked immunosorbent assay

The concentrations of tumor necrosis factor α (TNFα) and interleukin-6 (IL-6) in the samples were quantified using commercial enzyme-linked immunosorbent assay (ELISA) kits, according to the manufacturers’ protocols. In brief, standards and samples were added to the antibody-precoated microplates. After incubation and thorough washing, an enzyme-conjugated reagent was introduced. Following another incubation and washing cycle, a substrate solution was added for color development. The reaction was terminated with a stop solution, and the absorbance was immediately measured at 450 nm using a microplate reader. The analyte concentrations were determined by extrapolation from the respective standard curves.

### Masson staining

Following deparaffinization in xylene (I and II) and hydration through a graded ethanol series (100%, 95%, 85%, 70%), the paraffin sections were rinsed with distilled water. The nuclei were then stained with hematoxylin, differentiated with 1% acid alcohol, and blued in running water, followed by cytoplasmic staining with acid fuchsin. Subsequently, the sections were treated with phosphomolybdic/phosphotungstic acid solution, directly stained with aniline blue, and briefly rinsed with a weak acid solution to enhance contrast. Finally, the sections were rapidly dehydrated through a graded ethanol series, cleared in xylene, and mounted with neutral balsam.

### Activity of ALT, AST, BUN, and CRE

Serum alanine aminotransferase (ALT), aspartate aminotransferase (AST), blood urea nitrogen (BUN), and creatinine (CRE) levels were measured using commercially available kits according to the manufacturer’s standards (Nanjing Jiancheng Bioengineering Institute, China).

### RNA-sequencing analysis and NF-κB pathway

LSECs were treated under the following conditions: control, cocultured with steatotic 7702 cells, and cocultured with steatotic 7702 cells + SA-PEG-ALA. Transcriptomic profiling of each group was performed using RNA-seq. Subsequently, total protein was extracted from each group, and the expression levels of proteins associated with the NF-κB signaling pathway were detected by WB. In addition, IF staining was applied to observe the expression and localization of NF-κB p65 in each group.

### Synthesis of biotin-ALA

Lipoic acid (200 mg, 0.97 mmol, 1 equiv) was dissolved in dichloromethane (5 ml), followed by the addition of N-Boc-ethylenediamine (0.15 ml, 0.97 mmol, 1 equiv). Then, EDC hydrochloride (EDC·HCl, 280 mg, 1.46 mmol, 1.5 equiv) and DMAP (177 mg, 1.46 mmol, 1.5 equiv) were added sequentially. The reaction mixture was stirred at room temperature under nitrogen atmosphere overnight. Purification by silica gel column chromatography (petroleum ether/ethyl acetate) afforded compound 1. Compound 1 (100 mg, 0.29 mmol, 1 equiv) was dissolved in a mixture of dichloromethane (4 ml) and trifluoroacetic acid (1 ml) with stirring under nitrogen protection. The mixture was stirred at room temperature for 3 h. Acetonitrile was added, and the solution was evaporated to dryness to give the crude product, compound 2. Biotin (59 mg, 0.24 mmol, 1 equiv) was dissolved in *N*,*N*-dimethylformamide (DMF) with stirring in an oil bath at 343 K and then cooled to room temperature. Compound 2 (60 mg, 0.24 mmol, 1 equiv) was added, followed by the sequential addition of EDC·HCl (70 mg, 0.36 mmol, 1.5 equiv) and DMAP (44 mg, 0.36 mmol, 1.5 equiv). The reaction was stirred at room temperature under nitrogen atmosphere overnight. Purification by reverse-phase column chromatography (acetonitrile/water) yielded compound 3 (biotin-ALA).

### WB and co-immunoprecipitation

Liver tissues and cells were efficiently lysed with RIPA lysis buffer (89900, Thermo Fisher Scientific), supplemented with protease inhibitor cocktail (78445,Thermo Fisher Scientific). Total protein was extracted from the supernatant by centrifuging at 10,000*g* for 30 min at 4 °C, and the BCA Protein Assay Kit (23227, Thermo Fisher Scientific) was used to measure the concentration of protein. Protein separation was carried out using a 10% SDS-PAGE. Then, the proteins were transferred to PVDF membranes, which were blocked with 5% BSA in phosphate-buffered saline with Tween (PBST). The membranes were incubated with the primary antibody targeting the protein of interest overnight at 4 °C, followed by incubation with the relevant HRP-conjugated secondary antibody for 1 h at 37 °C. Finally, the bands were detected using the ECL kit (32209, Thermo Fisher Scientific). The antibodies utilized are detailed in Table S2. For co-immunoprecipitation (co-IP), the cells were incubated with prechilled IP lysis buffer for 5 min, then transferred into a 1.5-ml tube, and centrifuged at 12,000*g* for 15 min at 4 °C. The supernatant was collected, and the IP procedure was carried out following the protocol of the Pierce Co-IP Kit (88804, Thermo Fisher Scientific). WB was performed as previously described.

### Plasmid transfection and RNA interference

To up-regulate HSP70, overexpression plasmid vectors (GenePharma, Shanghai, China) were transfected with Lipofectamine 2000. After 48 h of transfection, the 293T cells were harvested for further experiments. HSP70-specific small interfering RNA (siRNA) was manufactured and obtained from GenePharma (Shanghai, China) with the following sequences: 5′-GGUCCUAAGAAUCGUUCAATT-3′ and 5′-UUGAACGAUUCUUAGGACCTT-3′. After the 293T cells reached 50% confluence, they were transfected with either control or targeted siRNA, according to the kit instructions.

### Cellular thermal shift assay

The cells were efficiently lysed with RIPA lysis buffer (89900, Thermo Fisher Scientific), supplemented with protease inhibitor cocktail (78445, Thermo Fisher Scientific). After lysis, the samples were centrifuged at 12,000 rpm for 10 min at 4 °C, and the supernatant was collected. The protein supernatant was equally divided into ten 1.5-ml EP tubes and subjected to heat treatment at different temperatures (37, 43, 49, 55, 61, and 66 °C) for 5 min. Immediately after the heat treatment, the samples were placed on ice to terminate the temperature-induced effects. After cooling, the samples were centrifuged at 12,000 rpm for 10 min at 4 °C, and the supernatant was collected again. Finally, 30 μl of loading buffer was added to the samples from each temperature-treated group for subsequent WB analysis to detect the changes in protein thermal stability.

### Statistical analysis

Data were expressed as the mean ± standard deviation (SD). Differences between multiple groups were compared using one-way analysis of variance (ANOVA). Differences between 2 groups were compared using 2-tailed Student’s *t* test. For comparisons between multiple groups, one-way ANOVA with a post hoc Tukey test was used. All analyses were performed using GraphPad Prism 10.0 software. When *P* values were <0.05, the differences were accepted as statistically significant.

## Results

### SELE was overexpressed in murine and human MASH

**Fig. 2.**
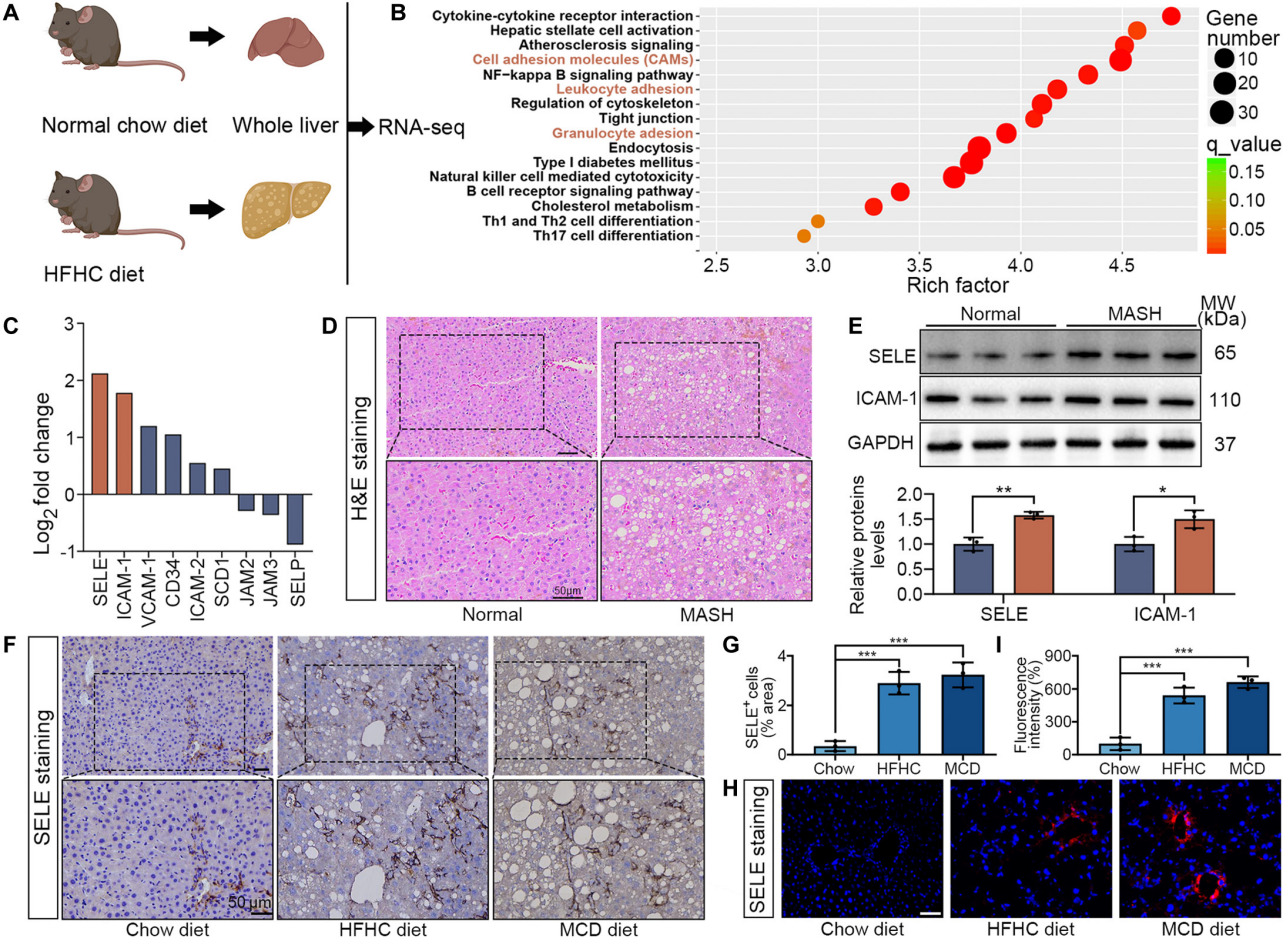
SELE was overexpressed in both murine and human MASH, with steatotic hepatocytes exacerbating endothelial dysfunction and promoting SELE expression in LSECs. (A) Schematic diagram illustrating the transcriptomic analysis of whole livers from 3 chow-fed and 3 HFHC-fed mice. (B) KEGG analysis of the enrichment pathways. Red font indicated pathways related to leukocyte adhesion. (C) Log_2_FC in the mRNA abundance of candidate genes encoding LSEC adhesion molecules in the livers of HFHC-fed versus chow-fed mice. (D) H&E staining was used to analyze both clinical normal and MASH liver samples. Scale bar, 50 μm. (E) Western blot (WB) was applied to detect the expression of *SELE* and *ICAM-1* in liver samples from MASH patients. (F and G) SELE immunohistochemical staining and quantification in diet-induced MASH model. Scale bar, 50 μm. (H and I) SELE immunofluorescent staining and quantification in diet-induced MASH model. Scale bar, 50 μm. Statistical significance was determined via a 2-tailed unpaired Student’s *t* test. Results are means ± SD of 3 independent experiments. ***P < 0.001.

Endothelial inflammation has been identified as an early hallmark of MASH, occurring prior to the infiltration of hepatic macrophages and the development of fibrosis [18]. To obtain a more comprehensive understanding of the pathophysiology of MASH, we conducted transcriptomic analyses using RNA-seq on whole livers of mice. Mice were fed either an HFHC diet or a normal chow diet for 20 weeks (*n* = 3 per each group) (Fig. 2A). We performed Kyoto Encyclopedia of Genes and Genomes (KEGG) pathway analysis on the up-regulated gene sets and identified that, among the top most significantly enriched canonical pathways, 3 were heavily implicated in leukocyte adhesion (Fig. 2B). This finding suggested a substantial pathophysiological role of leukocyte interaction with LSECs in driving the inflammatory response in MASH. Next we sought to identify the molecules involved in leukocyte adhesion to LSECs in the context of MASH. We identified “candidate genes” from the published literature that encode adhesion molecules on endothelial cells, which interacted with leukocyte adhesion molecules to mediate cell binding (Table S4) [19–21]. Among these candidate genes, we observed that *SELE* and *ICAM-1* were differentially up-regulated in the liver of MASH models (Fig. 2C). Additionally, we analyzed the Gene Expression Omnibus (GEO) database (GSE83452) and found that the expression of *SELE* was significantly elevated in the livers of MASH patients, while *ICAM-1* expression showed an increasing trend but did not reach statistical significance (Fig. S1A). Furthermore, the protein levels of SELE and ICAM-1 were also assessed in the liver tissues of MASH patients. WB analysis indicated that both SELE and ICAM-1 showed an increasing trend, with SELE exhibiting a more pronounced elevation (Fig. 2D and E). To validate the findings from the transcriptomic data and clinical samples, we assessed the mRNA expression levels of *SELE*. qPCR analysis revealed that *SELE* was markedly up-regulated in the livers of HFHC-fed mice compared to chow-fed controls (Fig. S1B and C). Besides, we explored the relationship between *SELE* mRNA expression and the non-alcoholic fatty liver disease activity score (NAS), and we observed a robust positive correlation between *SELE* levels and the NAS, suggesting that SELE may play a role in inflammatory progression (Fig. S1D). In addition, IHC and IF confirmed the elevated SELE protein expression in LSECs of HFHC-fed mice compared with chow-fed mice (Fig. 2F to I).

### Steatotic hepatocytes exacerbate endothelial dysfunction and promote the expression of SELE in LSECs

**Fig. 3.**
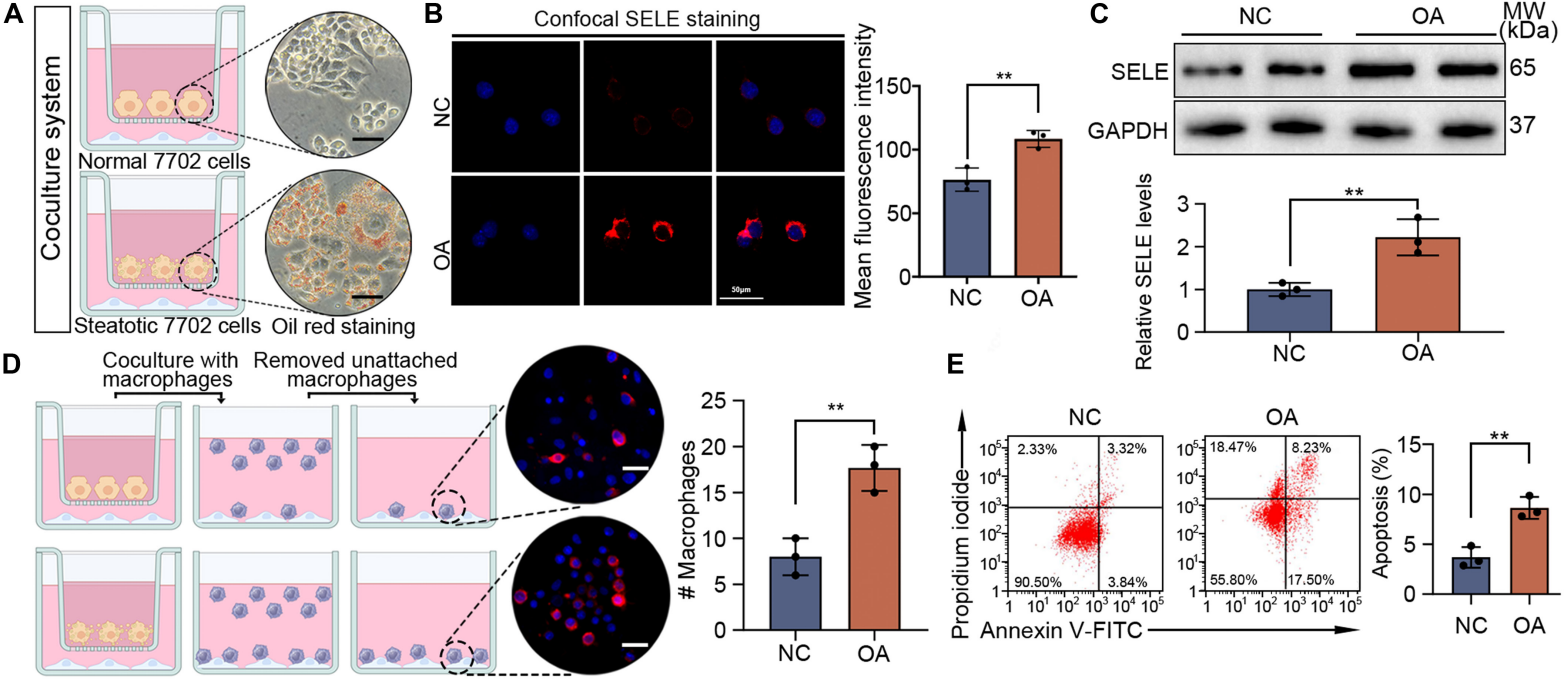
Steatotic hepatocytes exacerbating endothelial dysfunction and promoting SELE expression in LSECs. (A) Coculture schematic diagram of steatotic 7702 cells and LSECs. Scale bar, 30 μm. (B) SELE immunofluorescent staining in LSECs cocultured with steatotic 7702 cells. Scale bar, 20 μm. (C) WB was applied to detect the expression of SELE in LSECs cocultured with steatotic hepatocytes. (D) LSECs cocultured with steatotic 7702 cells, and quantification and images of adherent monocytes are presented. Scale bar, 30 μm. (E) Flow cytometry was applied for the analysis of apoptosis. Statistical significance was determined via a 2-tailed unpaired Student’s *t* test. Results are means ± SD of 3 independent experiments. **P < 0.01.

To further investigate the underlying reasons for the increased expression of SELE in LSECs in MASH, we hypothesized that fat accumulation in hepatocytes might promote SELE expression in LSECs. We cocultured oleic acid-induced steatotic 7702 cells with LSECs for 24 h and performed functional studies (Fig. 3A). Fluorescence staining and WB analysis demonstrated that LSECs treated with steatotic 7702 cells exhibited markedly higher SELE expression compared to those treated with normal 7702 cells (Fig. 3B and C). Furthermore, cell adhesion assays demonstrated that coculture with steatotic 7702 cells resulted in increased adhesion of monocytes to LSECs (Fig. 3D). Previous study showed that apoptosis played a critical role in cellular damage within tissues during inflammatory processes and disrupted endothelial function, manifesting that apoptosis was regarded as a secondary outcome of inflammation [22]. Hence, we assessed the apoptosis of LSECs following cocultured with steatotic 7702 cells. Flow cytometry confirmed increased apoptosis in LSECs treated with steatotic 7702 cells (Fig. 3E).

### Design, synthesis, and characterization of SA-PEG-ALA nanomicelles

**Fig. 4.**
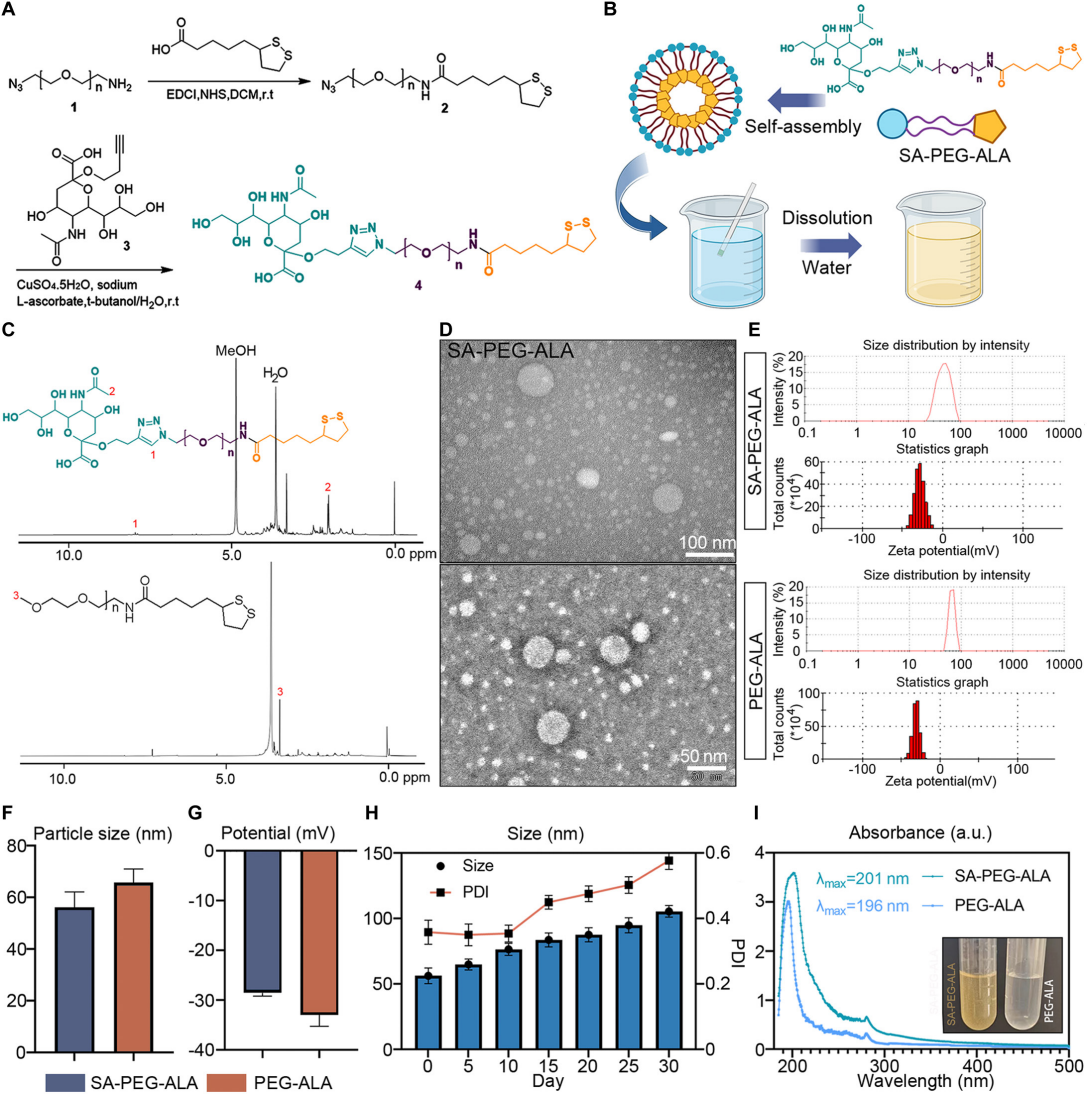
Design, synthesis, and characterization of SA-PEG-ALA nanomicelles. (A) Synthesis route of SA-PEG-ALA. (B) Preparation process of nanomicelles. (C) ^1^H NMR (400 MHz, MeOD) of SA-PEG-ALA and PEG-ALA. (D) TEM imaging of SA-PEG-ALA and PEG-ALA nanomicelles. (E) Particle size distribution and ζ-potential of SA-PEG-ALA and PEG-ALA nanomicelles. (F and G) Graphical statistics of particle size (nm) and potential (mV) of indicated nanomicelles. (H) Stability of SA-PEG-ALA nanomicelles during 30 d. (I) UV absorbance of SA-PEG-ALA and PEG-ALA nanomicelles. Results are means ± SD of 3 independent experiments.

Synthesis route of SA-PEG-ALA was shown in Fig. 4A and B. SA-PEG-ALA (210 mg), a pale yellow oily substance, was obtained with a yield of 30.0%. ^1^H nuclear magnetic resonance (NMR) (400 MHz, MeOD) δ 7.99 (d, J = 23.1 Hz, 1H), 4.63 to 4.55 (m, 2H), 4.06 to 3.97 (m, 2H), 3.91 (dq, J = 11.7, 6.1 Hz, 2H), 3.85 to 3.43 (m, 47H), 3.38 (td, J = 5.6, 3.0 Hz, 2H), 3.29 to 3.18 (m, 1H), 3.17 to 3.11 (m, 1H), 3.04 to 2.98 (m, 1H), 2.53 to 2.47 (m, 3H), 2.30 (d, J = 2.7 Hz, 1H), 2.29 to 2.22 (m, 1H), 2.07 (s, 3H), 2.04 (s, 6H), 1.75 to 1.60 (m, 4H), 1.31 (d, J = 3.4 Hz, 1H) (Fig. 4C). High-resolution electrospray ionization mass spectrometry (HRMS-ESI) calcd for C_(25+2n)_H_(41+4n)_N_5_O_(10+n)_S_2_ [M/2 + H]^+^ 582.78, 604.79, 626.80, etc., found 582.78, 604.79, and 626.80 (Fig. S2A). The important intermediates were illustrated in Fig. S2B and C.

The synthetic illustration of PEG-ALA was shown in Fig. S3A. PEG-ALA (1.7 g), a white substance, was obtained with a yield of 64.4%. ^1^H NMR (400 MHz, Chloroform-d) δ 3.62 (s, 250H), 3.47 to 3.41 (m, 3H), 3.35 (s, 4H), 3.21 to 3.00 (m, 2H), 2.79 (s, 1H), 2.49 to 2.36 (m, 1H), 2.17 (t, J = 7.4 Hz, 2H), 1.88 (dq, J = 12.8, 7.0 Hz, 1H), 1.71 to 1.55 (m, 3H), 1.52 to 1.35 (m, 2H), 1.23 (s, 1H) (Fig. 4C). HRMS (ESI) calcd for C_(13+2n)_H_(25+4n)_NO_(3+n)_S_2_ [M+2H]^+^ 638.9, 682.9, 726.9, etc., found 638.9, 682.9, and 726.9 (Fig. S3B).

The synthesis of SA-PEG was depicted in Fig. S4A. HRMS (ESI) calcd for C_(7+2n)_H_(13+4n)_N_3_O_(4+n)_ [M + H]^+^ 622.3, 666.3, 710.3, etc., found 622.3, 666.3, and 710.3 (Fig. S4B). HRMS (ESI) calcd for C_(22+2n)_H_(36+4n)_N_4_O_(13+n)_ [M + H]^+^, 959.5, 1,003.5, 1,047.5, etc., found 959.5, 1,003.5, and 1,047.5 (Fig. S4C). ^1^H NMR (400 MHz, Chloroform-d) δ 7.92 (s, 1H), 5.32 (d, J = 5.4 Hz, 2H), 4.20 (dd, J = 5.6, 3.5 Hz, 2H), 3.74 (d, J = 13.1 Hz, 3H), 3.59 (dtd, J = 18.8, 9.3, 4.1 Hz, 4H), 3.46 (s, 53H), 3.20 (s, 3H), 2.78 (d, J = 16.9 Hz, 2H), 2.29 (d, J = 7.0 Hz, 1H), 2.07 (s, 1H), 1.83 (d, J = 1.9 Hz, 3H), 1.44 (s, 1H) (Fig. S4D).

SA-PEG-ALA was dissolved in water to prepare SA-PEG-ALA nanomicelles, as shown in Fig. 4B. TEM observations revealed that both SA-PEG-ALA and PEG-ALA nanomicelles were spherical (Fig. 4D). The average particle size of SA-PEG- ALA nanomicelles was 58.32 ± 6.47 nm, the average zeta potential was −28.57 ± 0.67 mV, and the PDI was 0.36 ± 0.04. For PEG-ALA nanomicelles, the average particle size was 65.82 ± 5.23 nm, the average zeta potential was −33 ± 2.27 mV, and the PDI was 0.50 ± 0.09 (Fig. 4E to G). The SA-PEG-ALA nanomicelles exhibited good stability of nanoparticles in the first 10 d. After that, the nanoparticle size and PDI gradually increased (Fig. 4H). The maximum UV absorption of SA-PEG-ALA nanomicelles was at 201 nm, and that of PEG-ALA nanomicelles was at 196 nm, which as illustrated in Fig. 4I.

The in vitro drug release profiles of PEG-ALA and SA-PEG-ALA were evaluated using the dialysis method. Both micelles exhibited a typical biphasic release pattern, characterized by an initial burst release of approximately 30% within the first 4 h, followed by a sustained release phase. After 24 h, the cumulative drug release reached 53.3% for PEG-ALA and 54.8% for SA-PEG-ALA. While the release profiles of PEG-ALA and SA-PEG-ALA were similar, the free ALA diffused completely from the dialysis bag within 8 h (Fig. S4E).

### Evaluation of the targeting efficacy of SA-PEG-ALA in vitro

**Fig. 5.**
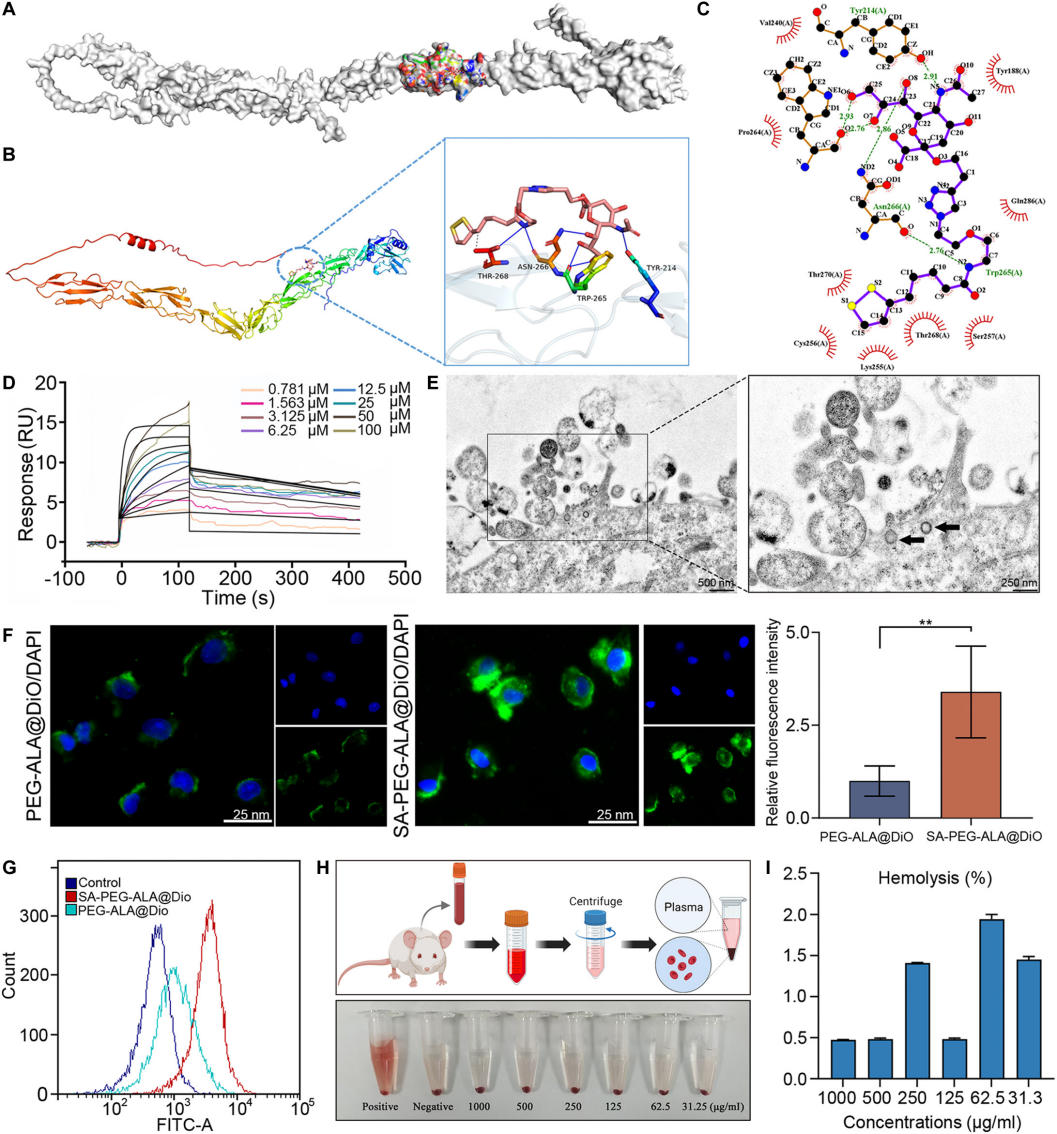
Evaluation of the targeting efficacy of SA-PEG-ALA in vitro*.* (A) Structure of SELE protein. (B) Analysis of 3-dimensional binding interactions between SA-PEG-ALA and SELE protein. (C) Analysis of 2-dimensional binding interactions between SA-PEG-ALA and SELE protein. (D) Binding curves of different concentrations of SA-PEG-ALA and SELE protein sensor chip by the SPR system. (E) Ultra-thin transmission electron microscopy image. Uptake of SA-PEG-ALA was shown as black arrow. Scale bar, 500 nm. (F) Confocal microscopy was used to detect the uptake of nanomicelles. Scale bar, 25 μm. (G) Cellular uptake of nanomicelles and evaluation of targeting properties using flow cytometry. (H) Hemolysis observation diagram. (I) Hemolysis ratio of different concentrations of SA-PEG-ALA. Statistical significance was determined via a 2-tailed unpaired Student’s *t* test. Results are means ± SD of 3 independent experiments. **P < 0.01.

To evaluate in vitro targeting efficiency of SA-PEG-ALA, we conducted molecular docking experiments between SA and SELE (Fig. 5A and B). The molecular docking results indicated that SA and SELE primarily interacted through hydrogen bonding and hydrophobic interactions. Specifically, SA formed hydrogen bonds with the amino acid residues Tyr^214^, Trp^265^, Asn^266^, and Thr^268^ of SELE. Additionally, the hydrophobic carbon chain of SA interacted with the hydrophobic amino acids Tyr^188^ and Pro^264^ of SELE (Fig. 5C and Table S5). Furthermore, we analyzed the binding energy of SA with SELE and predicted the dissociation constant (*K*_d_, nM). The results showed that the binding free energy (Δ*G*) was −3.31 kcal/mol, with a predicted dissociation constant of 3.74 nM, indicating a strong binding affinity between SA and SELE (Table S6). To further validate the molecular docking results and assess the binding strength between SA-PEG-ALA and SELE, we performed a surface plasmon resonance (SPR) experiment using SA-PEG-ALA and SELE. The dissociation equilibrium constant between SA-PEG-ALA and SELE was 4.16 × 10^−6^ nM, suggesting that SA-PEG-ALA has a strong affinity for the target SELE (Fig. 5D and Table S7). Next, we investigated the uptake of SA-PEG-ALA by LSECs. Ultrastructural TEM images revealed that LSECs were able to internalize SA-PEG-ALA nanomicelles, as indicated by the black arrows (Fig. 5E). To assess the in vitro targeting efficiency of SA-PEG-ALA, SELE overexpression in LSECs was induced by coculturing with steatotic 7702 cells. IF staining and flow cytometry experiments demonstrated that LSECs cocultured with steatotic 7702 cells were capable of internalizing both SA-PEG-ALA and PEG-ALA, with a significantly stronger uptake observed for SA-PEG-ALA (Fig. 5F and G). To further solidify the targeting mechanism, we applied competitive experiment for SELE. According to the competitive experiments, the addition of free SA significantly inhibited the uptake of SA-PEG-ALA, but not the uptake of nontargeted PEG-ALA (Fig. S5A to D). Consistent results were quantitatively validated by HPLC analysis (Fig. S5E). Moreover, we assessed the hemolytic activity of SA-PEG-ALA at different concentrations. The results showed that the hemolysis rate for all concentrations of SA-PEG-ALA was below 5%, indicating that intravenous tail injection was safe (Fig. 5H and I).

### Evaluation of the targeting efficacy of SA-PEG-ALA in vivo

**Fig. 6.**
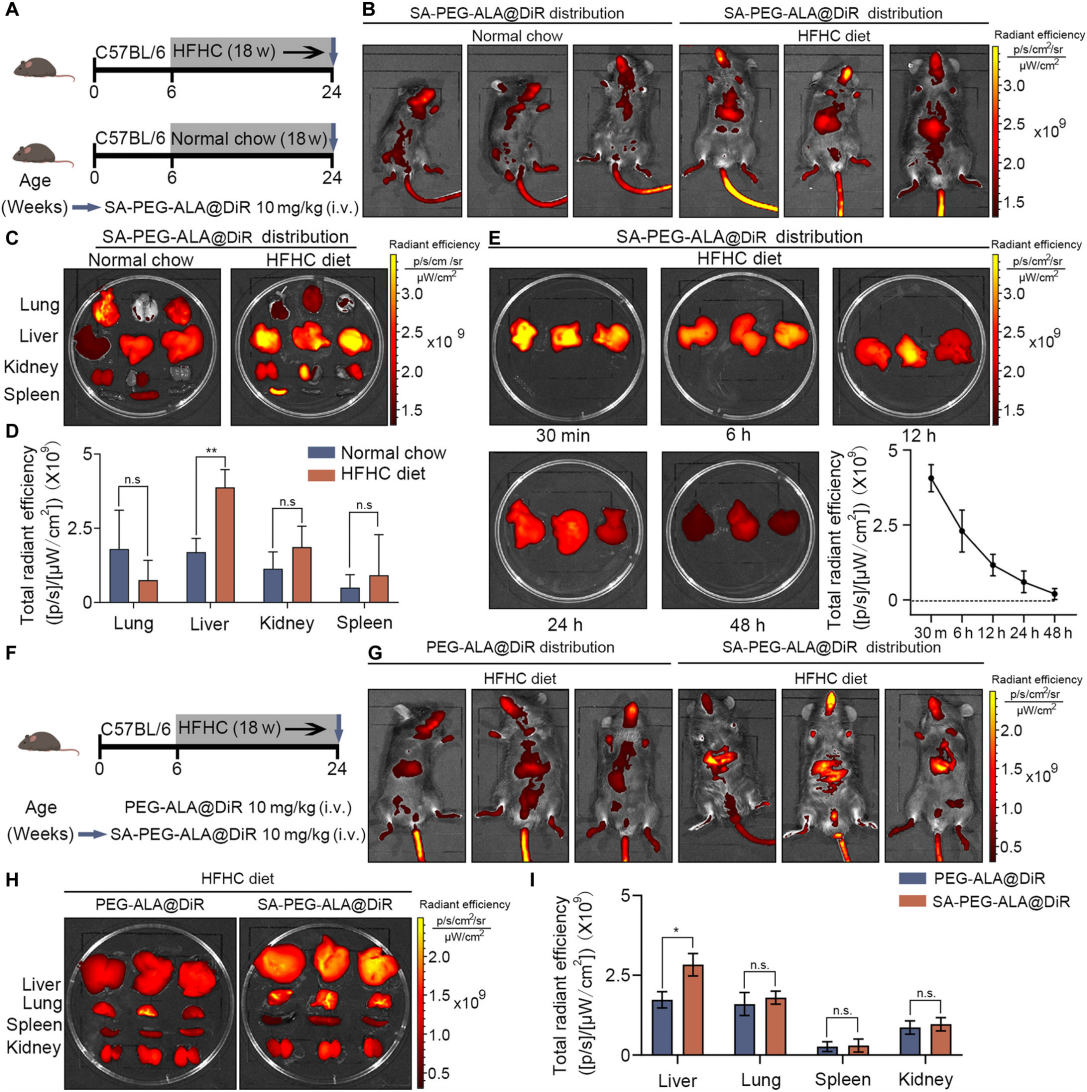
Evaluation of the targeting efficacy of SA-PEG-ALA in vivo*.* (A) Schedule of establishment of the MASH model. Mice received HFHC diet for 18 weeks. (B) SA-PEG-ALA@DiR nanomicelles were distributed mainly in the liver of MASH mice. (C and D) Ex vivo images and quantitative analysis of lung, liver, kidney, and spleen harvested from MASH or normal mice 30 min after administration of SA-PEG-ALA. (E) Ex vivo images and quantitative analysis of livers captured at 30 min, 6 h, 12 h, 24 h, and 48 h post-injection. (F) Schematic of the administration schedule for SA-PEG-ALA@DiR and PEG-ALA@DiR in MASH mice. (G) Representative in vivo fluorescence images of SA-PEG-ALA@DiR and PEG-ALA@DiR in MASH mice. (H and I) Representative ex vivo fluorescence images and corresponding quantification of the lung, liver, kidney, and spleen from MASH mice at 30 min post-injection of SA-PEG-ALA@DiR or PEG-ALA@DiR. Statistical significance was determined via a 2-tailed unpaired Student’s *t* test. Results are means ± SD of 3 independent experiments. *P < 0.05, **P < 0.01.

To assess the biodistribution of SA-PEG-ALA nanomicelles, SA-PEG-ALA@DiR was intravenously administered to HFHC diet-induced MASH mice after 18 weeks of feeding (Fig. 6A). At 30 min, 6 h, 12 h, 24 h, and 48 h post-tail vein injection, all mice were euthanized, and the fluorescence intensity of SA-PEG-ALA@DiR in the lung, liver, kidney, and spleen was quantified using a Xenogen IVIS Lumina system. Compared to normal mice, SA-PEG-ALA@DiR primarily accumulated in the liver of MASH mice within 30 min after intravenous administration, likely due to the targeting specificity of SA for the elevated SELE expression in the LSECs of MASH mice (Fig. 6B to D). In addition, the fluorescence of SA-PEG-ALA@DiR in the liver almost completely vanished within 48 h, implying that SA-PEG-ALA is gradually metabolized (Fig. 6E). Moreover, we performed a critical in vivo targeting experiment to directly compare the liver accumulation of the targeted SA-PEG-ALA versus the nontargeted PEG-ALA in HFHC-induced MASH mice (Fig. 6F). The results exhibited that the SA-PEG-ALA@DiR group exhibited a significantly stronger fluorescent signal in the liver compared to the PEG-ALA@DiR group at the same time point post-injection (Fig. 6G to I). To summarize, our results demonstrated that SA-PEG-ALA prepared in this study rapidly accumulated in the liver of MASH mice.

### SA-PEG-ALA attenuated steatosis and inflammation in an HFHC-induced MASH model

**Fig. 7.**
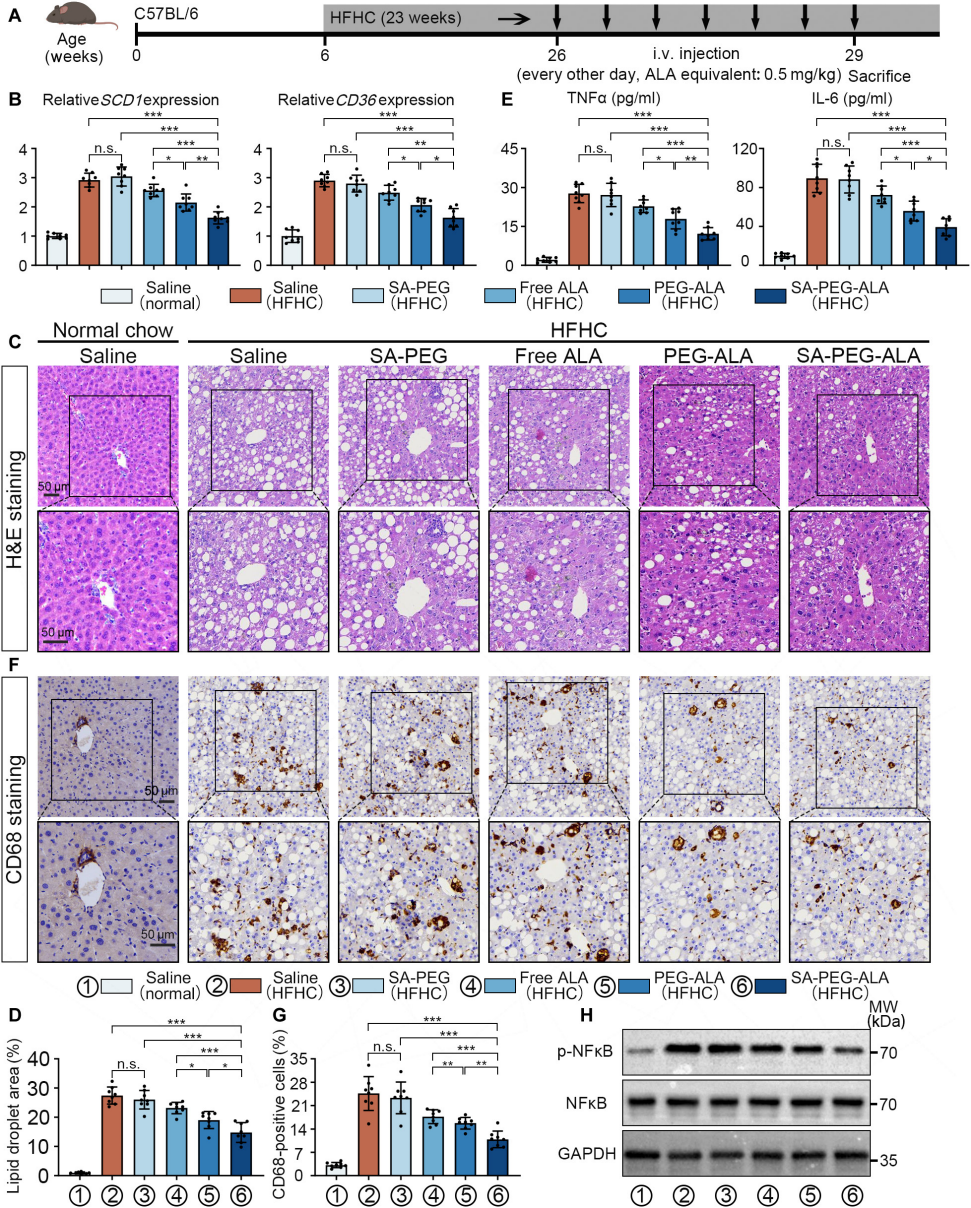
SA-PEG-ALA attenuated steatosis and inflammation in an HFHC-induced MASH model. (A) Schematic diagram of the HFHC-induced MASH model and the treatment timeline for saline, free ALA, SA-PEG, PEG-ALA, and SA-PEG-ALA. (B) Hepatic mRNA expression of lipid metabolism-related genes (*SCD1*, *CD36*) (*n* = 8 mice per group). (C and D) Representative microscopic images and quantitative analysis of H&E staining in the indicative groups (*n* = 8 mice per group). (E) ELISA assay detected the inflammatory cytokines in the indicative groups (*n* = 8 mice per group). (F and G) Representative microscopic images and quantitative analysis of CD68 staining in the indicative groups (*n* = 8 mice per group). (H) WBs of phosphorylation and total expression of NF-κB in the livers collected from the indicated groups (*n* = 8 mice per group). Statistical significance was determined via a one-way ANOVA with Tukey test. Results are means ± SD of 3 independent experiments. *P < 0.05, **P < 0.01, ***P < 0.001.

Based on our findings, we assessed the therapeutic efficacy of free ALA, SA-PEG, PEG-ALA, and SA-PEG-ALA micelles in a diet-induced MASH mice model (Fig. 7A). The gross images, body weight curves, body weight, liver weight, and liver-to-body weight ratio indicated a slight decrease in weight in the HFHC-fed mice treated with SA-PEG-ALA compared to the saline-treated MASH model mice (Fig. S6A to D). This might be attributed to the relatively short intervention period, as systemic metabolic changes may not yet be apparent. Therefore, we further assessed lipid metabolism and fat deposition in the liver tissue of each group. qPCR, H&E staining, Oil Red O staining, and quantitative analysis showed that both SA-PEG-ALA, PEG-ALA nanomicelles and free ALA improved liver tissue lipid metabolism and reduced lipid droplet deposition in the liver tissue after 4 weeks of treatment, and the most favorable therapeutic outcomes were observed with SA-PEG-ALA (Fig. 7B to D and Fig. S6E and F).

Moreover, ELISA assay, IHC, H&E staining, and quantitative analysis revealed that inflammatory cytokines (TNFα and IL-6), macrophage infiltration (CD68), and inflammatory foci in liver tissues were significantly increased in HFHC mice treated with saline or SA-PEG, compared to those of healthy mice fed a normal chow diet. Nanomicelles and free ALA intervention resulted in a reduction of inflammatory cytokines in serum and macrophage infiltration in liver tissues, with targeted SA-PEG-ALA nanomicelles therapy demonstrating a more significant effect compared to nontargeted PEG-ALA nanomicelles and free ALA (Fig. 7E to G and Fig. S6G). Consistent with the CD68 staining results, SA-PEG-ALA nanomicelle treatment most efficiently reduced liver inflammatory levels in HFHC mice compared to all other treatment groups (Fig. 7H).

### SA-PEG-ALA ameliorated fibrosis and liver injury in an HFHC-induced MASH model

**Fig. 8.**
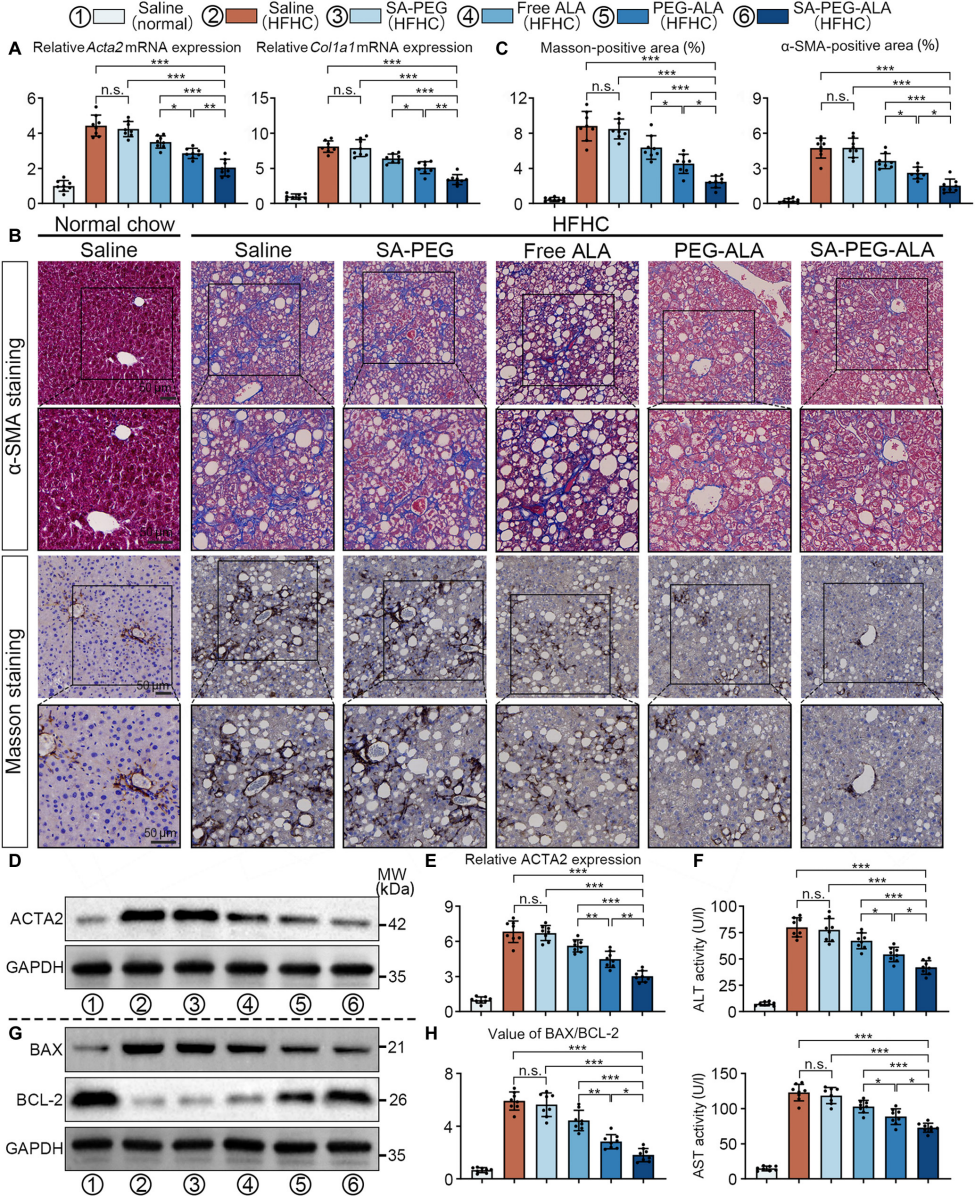
SA-PEG-ALA ameliorated fibrosis and liver injury in an HFHC-induced MASH model. (A) Hepatic mRNA expression of profibrogenic genes (*ACTA2*, *COL1A1*) (*n* = 8 mice per group). (B and C) Representative microscopic images and quantitative analysis of Masson’s trichrome and αSMA staining in the indicative groups (*n* = 8 mice per group). (D and E) Western blots and quantitative analysis of ACTA2 in the livers collected from the indicated groups (*n* = 8 mice per group). (F) Serum ALT and AST levels of mice receiving the indicated treatments (*n* = 8 mice per group). (G and H) WBs and quantitative analysis of BAX and BCL-2 in the livers collected from the indicated groups (*n* = 8 mice per group). Statistical significance was determined via a one-way ANOVA with Tukey test. Results are means ± SD of 3 independent experiments. *P < 0.05, **P < 0.01, ***P < 0.001.

Hepatic stellate cells (HSCs) played a crucial role in the initiation and progression of MASH and progression [23]. This process resulted in the up-regulation of *ACTA2* and *COL1A1* expression and enhanced the production of collagen. We applied qPCR analysis and found that delivery of ALA by nanomicelles obviously reduced the elevated expression levels of *ACTA2* and *COL1A1* induced by HFHC diet. Among the experimental groups, SA-PEG-ALA targeted delivery of ALA to LSECs exhibited the greatest efficacy in suppressing the expression of profibrogenic genes (Fig. 8A). Subsequently, Masson’s trichrome and αSMA staining analysis illustrated that HFHC diet induced noticeable liver fibrosis, as evidenced by accumulated aniline blue- and αSMA-positive area in liver tissues, all of which were attenuated in mice treated with SA-PEG-ALA, PEG-ALA, or free ALA. Explicitly, HFHC mice receiving SA-PEG-ALA treatment exhibited the least amount of fibrotic tissue among all the groups (Fig. 8B and C). Consistent with the qPCR, Masson’s trichrome, and IHC staining, WB analysis revealed that the up-regulation of ACTA2 in the livers of HFHC mice was significantly inhibited following treatment with nanomicelles or free ALA. In a similar fashion, HFHC mice treated with SA-PEG-ALA nanomicelles displayed the lowest expression levels of ACTA2 in the liver tissues (Fig. 8D and E).

Furthermore, compared to mice fed a normal chow diet, those on the HFHC diet exhibited noteworthy liver damage, as confirmed by elevated serum levels of ALT and AST. All these hepatic lesion characteristics were ameliorated in mice treated with free ALA, nontargeted PEG-ALA nanomicelles, or targeted SA-PEG-ALA nanomicelles, with the most effective therapeutic outcome attained by SA-PEG-ALA (Fig. 8F). Hepatic lesion also leads to the abnormal expression of apoptosis-related proteins, such as Bax and Bcl-2, with Bax acting as a pro-apoptotic protein and Bcl-2 causing the opposite effect as an anti-apoptotic protein. The alterations in the expression of Bax and Bcl-2 may potentially trigger mitochondrial outer membrane permeabilization, which in turn activates apoptosis. In line with the serological analysis, treatment with SA-PEG-ALA was the most effective in lowering pro-apoptotic protein Bax and boosting anti-apoptotic protein Bcl-2 (Fig. 8G and H). These results suggested that targeted delivery of ALA to LSECs effectively inhibited collagen deposition, liver fibrosis, and liver damage in the MASH model.

The biological safety of nanomicelles was assessed through pathological section analysis in HFHC-induced mice following the final administration. As illustrated in Fig. S7A to C, neither of the applied nanomicelles or free ALA therapeutics resulted in pathological abnormalities in the major organs (including the heart, spleen, lung, and kidney) nor caused notable changes in organ coefficient, BUN, or serum CRE when compared to the healthy controls, indicating minimal systemic toxicity throughout the treatment period. The 3-(4,5-dimethylthiazol-2-yl)-2,5-diphenyltetrazolium bromide (MTT) assay also indicated that the synthesized drug has no toxic side effects on the LSECs (Fig. S7D).

### SA-PEG-ALA inhibited steatotic hepatocyte-induced endothelial dysfunction

**Fig. 9.**
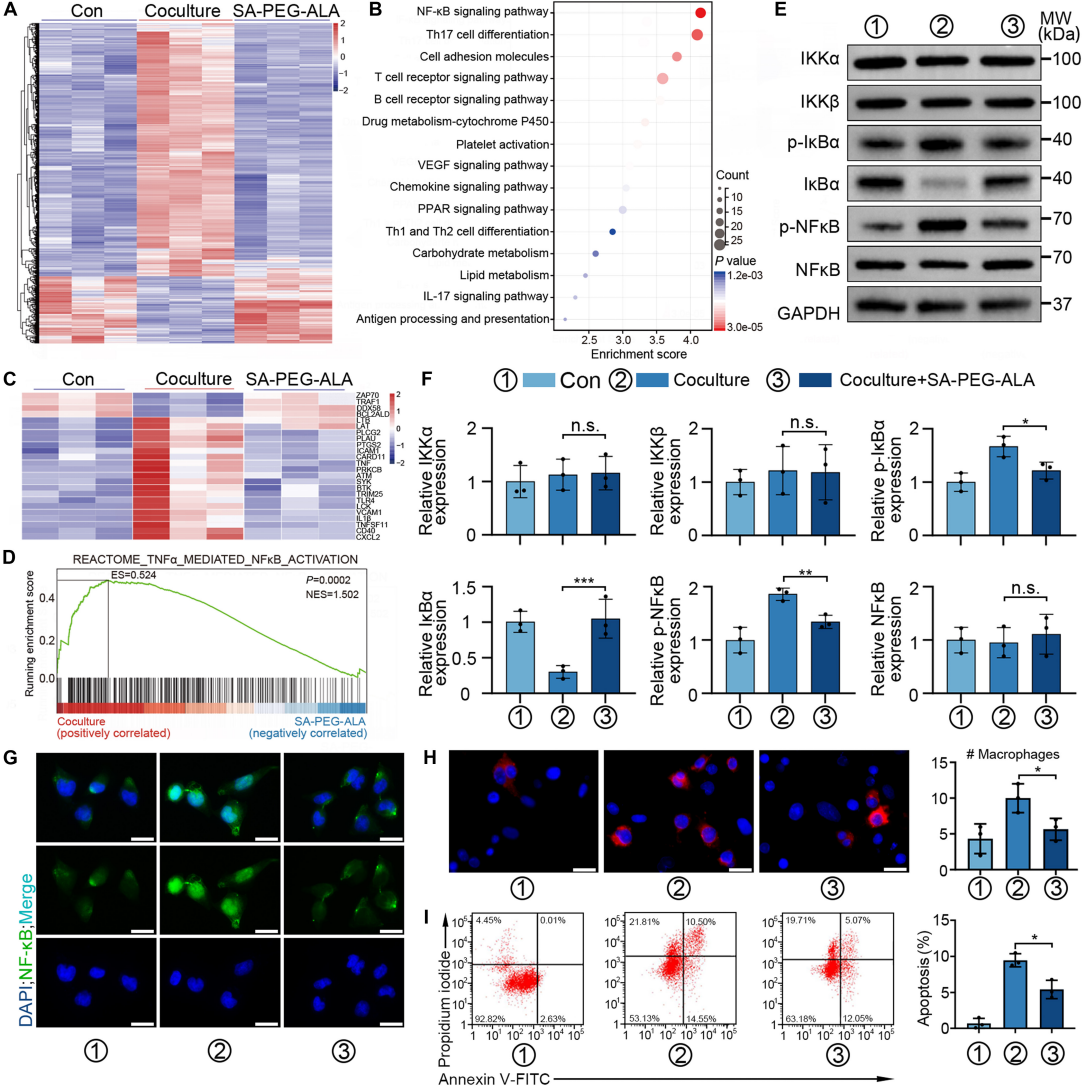
SA-PEG-ALA inhibited steatotic hepatocyte-induced endothelial dysfunction. (A) Heat map representing the significantly regulated genes detected in RNA-seq analysis of LSECs in the indicative groups. (B) KEGG pathway enrichment analysis in LSECs cocultured with steatotic 7702 cells compared with control. (C) Heat map representing the NF-κB signaling pathway-related genes detected in RNA-seq analysis of LSECs. (D) Gene set enrichment analysis showed that the NF-κB signaling pathway was negatively correlated in the SA-PEG-ALA treatment group. (E and F) WBs and quantitative analysis of IκBα/NF-κB signaling molecules in LSECs in the indicated groups. (G) IF staining was applied for the analysis of NF-κB p65 translocation in LSECs in the indicated groups. (H) Representative images and quantification of adherent monocytes to LSECs in the indicated groups. (I) Flow cytometry and quantitative analysis were applied for the analysis of apoptosis in LSECs in the indicated groups. Statistical significance was determined via a one-way ANOVA with Tukey test. Results are means ± SD of 3 independent experiments. *P < 0.05, **P < 0.01, ***P < 0.001.

To investigate the molecular mechanisms responsible for the protective effects of SA-PEG-ALA on LSECs, RNA-seq was conducted on LSECs from 3 groups: control, cocultured with steatotic 7702 cells, and the coculture group with SA-PEG-ALA intervention. The differentially expressed genes (DEGs) for the specified comparison groups were shown in Fig. 9A and Fig. S8A and B. According to the KEGG analysis , the NF-κB signaling pathway was the most prominently enriched among the DEGs in the coculture group compared to the control group (Fig. 9B). In contrast, SA-PEG-ALA remarkably diminished the enrichment of NF-κB signaling pathway-related DEGs induced by coculturing with steatotic 7702 cells (Fig. 9C). Furthermore, gene set enrichment analysis (GSEA) validated that NF-κB signaling pathway was up-regulated in coculture group and down-regulated by SA-PEG-ALA, suggesting that SA-PEG-ALA may exert protective effects against coculturing with steatotic 7702 cells through the inhibition of NF-κB signaling pathway (Fig. 9D). Consistent with the results of RNA-seq data, coculturing with steatotic 7702 cells obviously increased the phosphorylation of IκBα and NF-κB, and IKKα/β, without altering their total protein levels. In contrast, the results were reversed in LSECs following SA-PEG-ALA application (Fig. 9E and F). Fluorescence staining revealed that LSECs exposed to steatotic 7702 cells displayed prominent nuclear NF-κB staining; however, those treated with SA-PEG-ALA exhibited minimal nuclear NF-κB staining, implying NF-κB activation and translocation (Fig. 9G). In addition, WB analysis, adhesion assays, and flow cytometry experiments demonstrated that treatment with SA-PEG-ALA effectively reduced the expression of SELE and the number of monocyte adhesion and lowered the level of apoptosis in LSECs induced by coculturing with steatotic 7702 cells (Fig. 9H and I and Fig. S8C). Taken together, these results showed that SA-PEG-ALA potently inhibited steatotic hepatocyte-induced endothelial dysfunction via NF-κB signaling pathway.

### SA-PEG-ALA mediates the interaction between HSP70 and IκBα

**Fig. 10.**
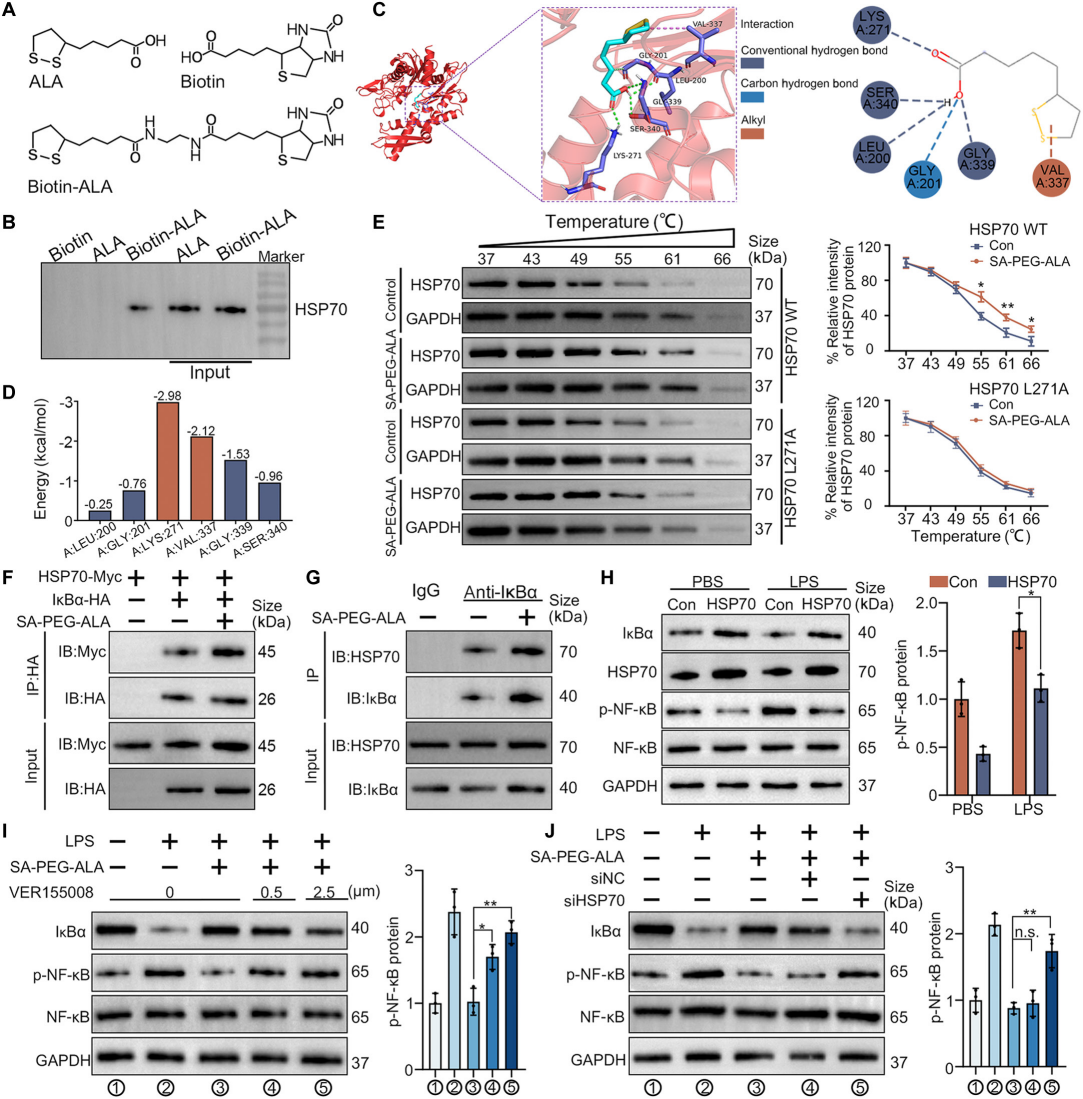
SA-PEG-ALA mediates the interaction between HSP70 and IκBα. (A) Chemical structure of ALA and biotin-ALA. (B) The pull-down assays exhibited that biotin-ALA bound to HSP70 in the LSECs lysates. (C) Molecular docking performed the binding pose details between ALA and HSP70. (D) Histograms of the per-residue energy contributions of key residues involved in ALA and HSP70. (E) Cellular thermal shift assay between ALA and HSP70. The curve was fitted using GraphPad Prism 10.0 (*n* = 3 per group). (F) HEK293T cells expressing specified protein were treated with SA-PEG-ALA (100 μg/ml) for 24 h. IP was then conducted using HA tag, followed by WB with a Myc antibody to detect the interaction between HSP70 and IκBα. (G) LSECs were treated with SA-PEG-ALA (100 μg/ml) for 24 h. Endogenous IP was conducted with an anti-IκBα antibody, followed by WB with an HSP70 antibody to detect the HSP70–IκBα interaction. (H) WB and quantitative analysis were applied to detect that HSP70 overexpression reversed the increase in NF-κB phosphorylation levels induced by LPS treatment. (I) HEK293T cells pretreated with LPS were cotreated with SA-PEG-ALA (100 μg/ml) and a specified dosage of VER155008. WB and quantitative analysis were applied to detect the expression of p-NF-κB. (J) HEK293T were transfected with siNC or siHSP70 and treated with LPS or SA-PEG-ALA (100 μg/ml). WB and quantitative analysis were applied to detect the expression of p-NF-κB. Data in (E) and (H) were determined via a 2-tailed unpaired Student’s *t* test. Data in (I) and (J) were determined via a one-way ANOVA with Tukey test. Results are means ± SD of 3 independent experiments. *P < 0.05, **P < 0.01.

To uncover the mechanism through which SA-PEG-ALA interfered steatotic hepatocyte-induced endothelial dysfunction, the biotin-ALA was synthesized and employed to identify the potential target of ALA (Fig. 10A and Fig. S9A to C). The chemical structure of biotin-ALA was characterized using ^1^H NMR (400 MHz, Chloroform-d), as presented in Fig. S9D and E. The previous study has reported that heat shock protein 70 (HSP70) played a crucial role in cellular stress responses and was involved in regulating various inflammatory signaling pathways to protect against acute liver injury [24,25]. We hypothesized that ALA might exert its effect in inhibiting the NF-κB signaling pathway by interacting with HSP70. To confirm whether ALA bound to HSP70 in cell lysates, biotin-ALA was applied to streptavidin-agarose beads and co-incubated with lysates of LSECs. The pull-down assays exhibited that biotin-ALA bound to HSP70 in the LSECs lysates (Fig. 10B). Subsequently, we conducted protein structure-based molecular docking and dynamics simulations to explore how ALA interacted with HSP70. According to the docking results, ALA formed extensive conventional hydrogen bonds, carbon hydrogen bonds, and alkyl to interact with HSP70. As the dynamics simulations revealed, the key residue involved in the interaction between ALA and HSP70, which exhibited the top average energy values, was Lys^271^ (Fig. 10C and D). To confirm the findings from molecular docking and dynamics simulations, we performed a cellular thermal shift assay (CETSA). As illustrated in Fig. 10E, ALA demonstrated a physical interaction with HSP70. However, the mutation of Lys^271^ in HSP70 substantially impaired the interaction. In conclusion, ALA could directly interact with HSP70.

Given the role of HSP70 in maintaining IκBα kinase activity, we designed this study to investigate the interaction between ALA and the HSP70/IκBα proteins. We conducted a co-IP assay using IκBα-HA (hemagglutinin) precipitation and found that the interaction between HSP70 and IκBα was augmented after SA-PEG-ALA induction in 293T cells (Fig. 10F). Besides, endogenous co-IP analysis further confirmed that the binding affinity between HSP70 and IκBα was enhanced by SA-PEG-ALA treatment in LSECs (Fig. 10G). To further investigate the effect of HSP70 on NF-κB phosphorylation, 293T cells were transfected with either an HSP70 overexpression plasmid or a control plasmid and subsequently stimulated with lipopolysaccharide (LPS), where HSP70 overexpression reversed the increase in NF-κB phosphorylation levels induced by LPS treatment (Fig. 10H). Additionally, the decrease in phosphorylated NF-κB and the increase in IκBα expression induced by SA-PEG-ALA treatment were reversed by VER155008, an inhibitor of HSP70 (Fig. 10I). Consistent results were observed, showing that SA-PEG-ALA had minimal impact on the down-regulation of the NF-κB signaling pathway in 293T cells with low HSP70 expression (Fig. 10J). Together, SA-PEG-ALA inhibited the NF-κB signaling pathway by promoting the interaction between HSP70 and IκBα.

## Discussion

Currently, patients with simple steatosis face a relatively low risk for adverse outcomes; however, this risk can escalate rapidly as the condition progresses to MASH [26]. The precise mechanisms underlying the progression from steatosis to MASH remain incompletely understood. Several key elements, including hepatic lipotoxicity, chronic inflammation, and endothelial dysfunction, have been identified as influencing the transition from fatty liver to MASH [27,28]. Among the various factors contributing to MASH progression, the dysfunction of LSECs has emerged as a critical and somewhat underexplored mechanism. LSECs, in physiological conditions, were recognized for their anti-inflammatory role; however, in the context of sustained inflammation, LSECs adopted a pro-inflammatory phenotype and function, which triggered the recruitment of immune cells and the exacerbation of hepatocyte injury [29]. One of the mechanisms underlying the phenotypic transition of LSECs was the release of integrin β1-enriched extracellular vesicles from hepatocytes under lipotoxic stress, which promoted monocyte adhesion to LSECs, thereby facilitating hepatic inflammation in MASH [30]. Combined with sequencing and experimental data, we found that LSECs in MASH indicated abnormal expression of SELE. Specifically, we also observed that steatotic hepatocytes induce both the up-regulation of SELE in LSECs and the onset of LSEC dysfunction. Although the potential mechanisms remain to be explored, these findings suggest that LSECs not only respond to liver inflammation but actively participate in its propagation, highlighting their potential as a therapeutic target in preventing or mitigating the progression of MASH.

SELE, a member of the selectin family, is primarily expressed in endothelial cells, including LSECs, and plays a key role in mediating the adhesion and transmigration of leukocytes from the bloodstream to tissues in response to inflammatory signals. SELE-mediated inflammation has been observed in alcohol-related liver disease, where its overexpression contributed to neutrophil infiltration and liver injury [31]. The use of SELE binding polymers has been investigated as a therapeutic approach to alleviate hepatic injury. In the context of MASH, soluble SELE levels have been associated with the disease severity, suggesting its potential as a biomarker for liver inflammation [32]. Additionally, recent study has provided compelling evidence that blocking the SELE-mediated neutrophil adhesion, driven by its high expression in adipose tissues, could significantly reduce liver inflammation and fibrosis in animal models of MASH [33]. Overall, SELE is a pivotal molecule in the inflammatory processes of the liver, making it a potential therapeutic target for reducing inflammation and preventing further liver damage.

SA, a naturally occurring monosaccharide found on the surface of glycoproteins and glycolipids, has long been recognized for its ability to interact with selectins. This interaction has prompted significant interest in utilizing SA as a targeting molecule for various therapeutic strategies. Previous studies have demonstrated that the modification of drug delivery systems with SA not only enhanced targeting of cancer cells but also helped evade the reticuloendothelial system, a major barrier to the clinical application of nanoparticle-based therapies [34]. SA-coated nanoparticles have shown reduced interactions with the innate immune system, leading to increased tumor accumulation and improved therapeutic efficacy [35]. Moreover, an SA-modified doxorubicin hydrochloride liposome has been shown to effectively target peripheral blood neutrophils (PBNs). This innovative approach induced apoptosis in PBNs by binding to SELL, a molecule highly expressed on activated PBNs, while also alleviating the neutrophil-related inflammation [36,37]. Based on these findings, we hypothesized that SA-modified nanomicelles might effectively target dysfunctional LSECs that overexpressed SELE. At the molecular level, we first confirmed the interaction between SA and SELE, which was established through molecular docking and SPR techniques. These results revealed that SA bound efficiently to SELE with favorable binding energies. Building on the molecular-level confirmation, we then examined the targeting efficiency of SA-PEG-ALA at the cellular level. Using a coculture model of steatotic hepatocytes and LSECs, we assessed the internalization of SA-PEG-ALA by confocal microscopy and flow cytometry. Compared to LSECs with low SELE expression, those with high SELE expression were more prone to internalizing SA-PEG-ALA. To assess the targeting specificity of SA-PEG-ALA in vivo, we conducted experiments in an HFHC-induced MASH animal model. In vivo imaging revealed that SA-PEG-ALA nanomicelles predominantly accumulated not only in liver but also in brain, further indicating that SA-PEG-ALA can specifically target endothelial cells in various organs. We hypothesized that this was likely due to MASLD causing neuropathological changes, including vascular damage and cerebellar atrophy, and neurodegeneration [38]. Together, these results underscored the potential of SA-PEG-ALA as a highly targeted drug delivery system, particularly for treating MASH.

Then, the therapeutic efficacy of SA-PEG-ALA was evaluated in vivo. SA-PEG-ALA demonstrates significant therapeutic efficacy in treating MASH, as evidenced by its ability to reduce liver inflammation, inhibit fibrosis, and protect against hepatocyte injury, possibly due to targeting distribution and combinational effect of ALA. Despite that the promising therapeutic potential of ALA has been reported before, several challenges remained in its clinical application [39]. One of the main drawbacks of ALA therapy is its poor bioavailability due to rapid metabolism, which may be caused by the extensive activation of neutrophils in MASH liver tissue, releasing elastase and proteinase 3 that quickly degrade other drug molecules [40]. Additionally, ALA’s hydrophobic nature limits its solubility and stability in aqueous environments, making it difficult to deliver effectively to target tissues. To address the limitations of ALA therapy, we conjugated ALA to an SA-modified PEG polymer, enhancing the solubility and stability of ALA in aqueous environments and ensuring sustained release and targeted delivery to the liver. Among all the groups, our H&E staining, IHC staining, and Masson’s trichrome staining results indicated that SA-PEG-ALA demonstrated superior therapeutic effects compared to the other groups.

Finally, to explore the molecular mechanisms through which SA-PEG-ALA exerted its effects, we first conducted RNA-seq in LSECs treated with SA-PEG-ALA. Our results indicated that SA-PEG-ALA effectively inhibited the activation of the NF-κB pathway in LSECs, a critical signaling pathway involved in inflammation and endothelial dysfunction [41–43]. It is reported that NF-κB played a central role in mediating the inflammatory response by up-regulating the expression of pro-inflammatory cytokines and adhesion molecules, which contribute to immune cell recruitment and tissue damage [44,45]. To further elucidate the mechanism by which ALA inhibited the NF-κB pathway, we innovatively tagged ALA with biotin. This modification allowed us to track ALA’s interactions at the molecular level. Applying pull-down assay and molecular docking, we discovered that ALA directly interacted with HSP70, a key molecular chaperone involved in regulating protein stability and cellular stress responses [46]. In addition, thermal shift assay revealed that ALA significantly enhanced the stability of HSP70, which may be critical for its ability to modulate inflammatory signaling pathways. To validate the role of HSP70 in SA-PEG-ALA’s therapeutic effects, we used VER155008, a specific HSP70 inhibitor, to block the interaction between ALA and HSP70 [47]. VER155008 has previously been used to study HSP70’s role in trophoblast survival during human placentation [48]. In our study, the application of VER155008 significantly weakened the inhibitory effect of SA-PEG-ALA on the NF-κB signaling pathway, indicating that HSP70 had a pivotal role in the anti-inflammatory effects of SA-PEG-ALA. Consistent results were observed when HSP70 expression was knocked down using siRNA.

In summary, our study provided compelling evidence that SA-PEG-ALA manifested its therapeutic effects in MASH by inhibiting the NF-κB signaling pathway, primarily through its interaction with HSP70. Our study not only enhances our understanding of the molecular mechanisms driving SA-PEG-ALA’s effects but also lays a solid foundation for its future clinical application in MASLD.

In conclusion, we designed a PEG-based nanomicelle system that incorporated SELE targeting functionality and offered hepatoprotective effects. After undergoing hydrophobic modification with PEG and targeting modification with SA, the resulting SA-PEG-ALA was capable of self-assembling into nanomicelles, effectively linking hydrophobic ALA, and facilitating sustained drug release. By virtue of the specific interaction between SA and SELE, SA-PEG-ALA was able to selectively internalize into LSECs cocultured with steatotic 7702 cells, enabling targeted distribution to the lesion sites in MASH mice. Both SA-PEG-ALA and PEG-ALA exhibited hepatoprotective effects; however, in vivo studies demonstrated that SA-PEG-ALA was the most effective in mitigating steatosis, inflammation, fibrosis, and liver injury in HFHC-induced MASH model. In addition, this study is the first to demonstrate that ALA sustains its therapeutic effects in improving MASH by inhibiting the NF-κB signaling pathway through HSP70. The findings from our study indicate that the multifunctional SA-PEG-ALA holds great potential as an effective delivery system for MASLD treatment.

## Data Availability

The datasets used and/or analyzed during the current study are available from the corresponding author upon reasonable request.
