# Supplementary figures and images for: E-Selectin-Targeted Nanomicelles via Sialic Acid Conjugation for Anti-Inflammatory Efficacy and Alleviating the Progression of Metabolic-Associated Steatotic Liver Disease
Source: Biomater Res. 2026 Feb 2;30:0305. doi: 10.34133/bmr.0305 (PMC12862626; doi:10.34133/bmr.0305)

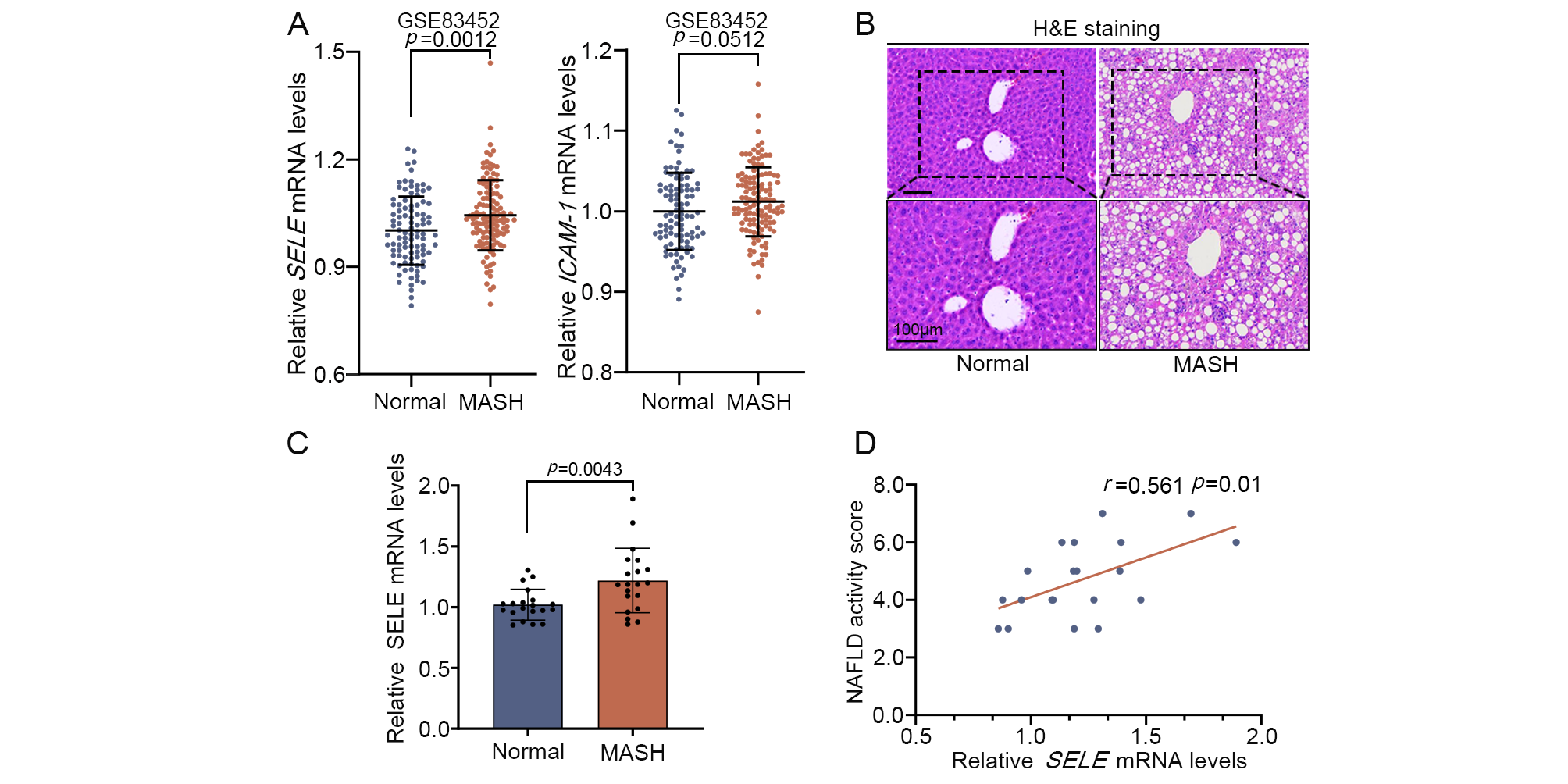

Supplement: Supplementary 1 — Figs. S1 to S9 Tables S1 to S7 [file bmr.0305.f1.zip › supplymentary_figure1.tif]

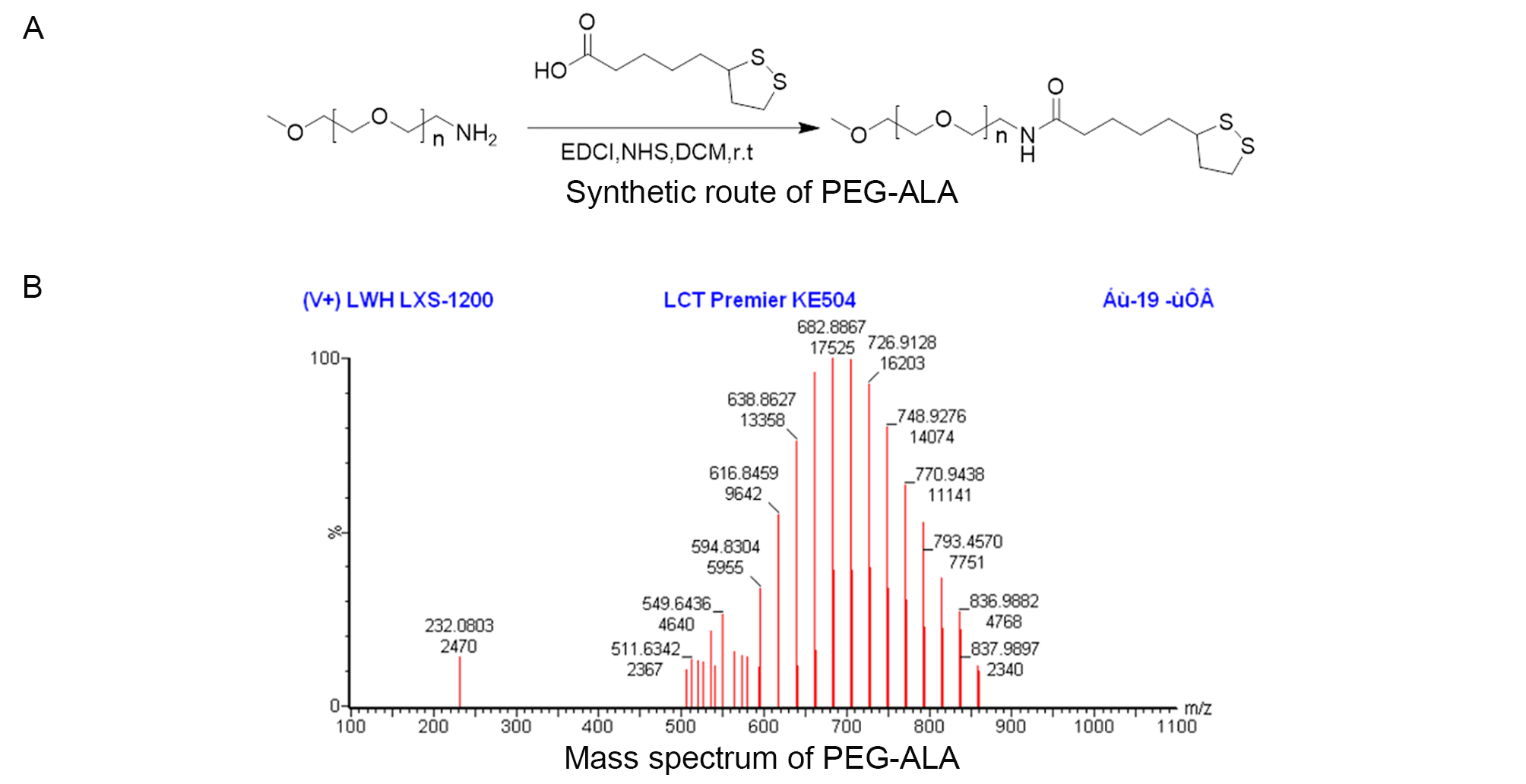

Supplement: Supplementary 1 — Figs. S1 to S9 Tables S1 to S7 [file bmr.0305.f1.zip › supplymentary_figure3.tif]

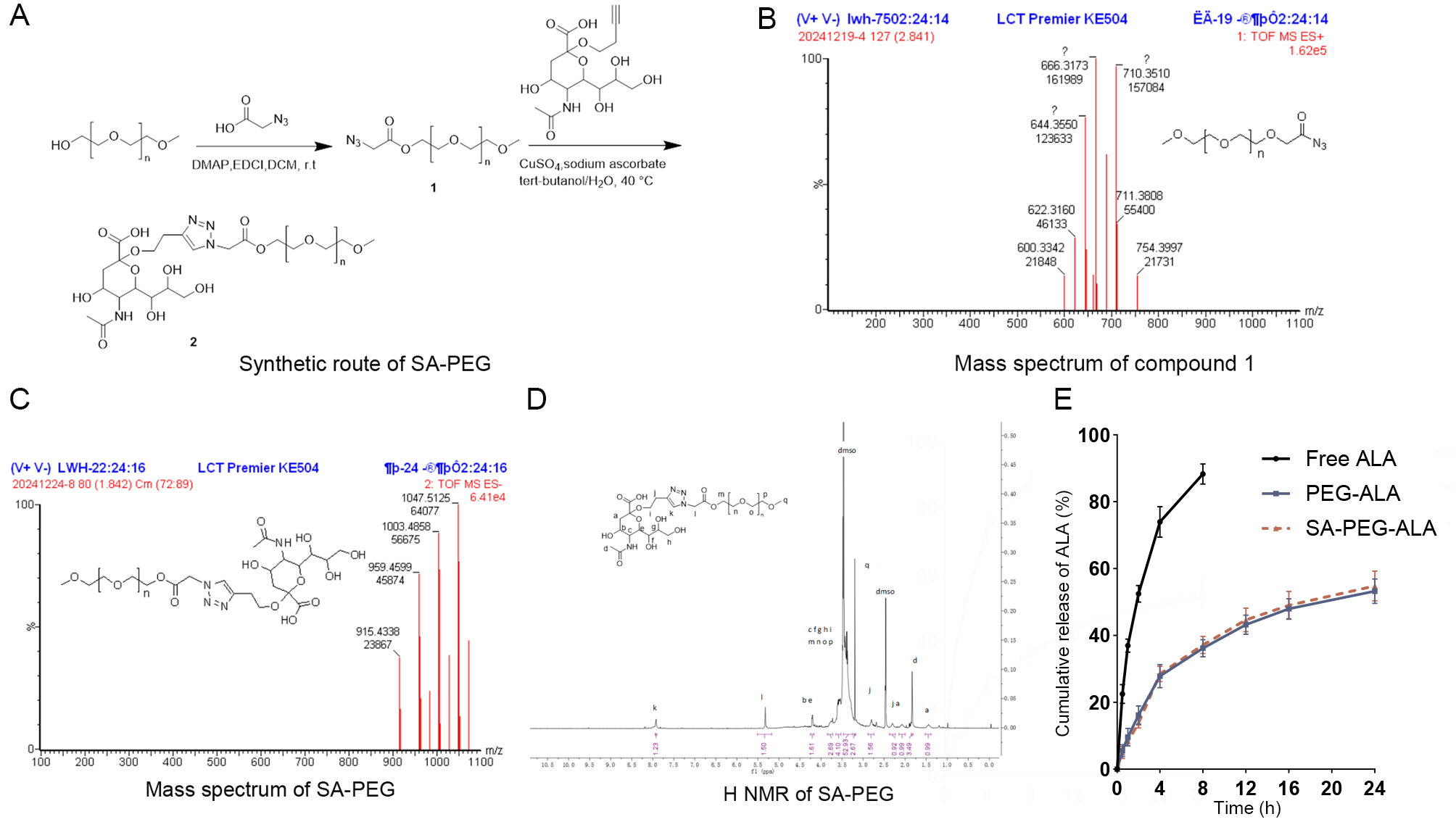

Supplement: Supplementary 1 — Figs. S1 to S9 Tables S1 to S7 [file bmr.0305.f1.zip › supplymentary_figure4.tif]

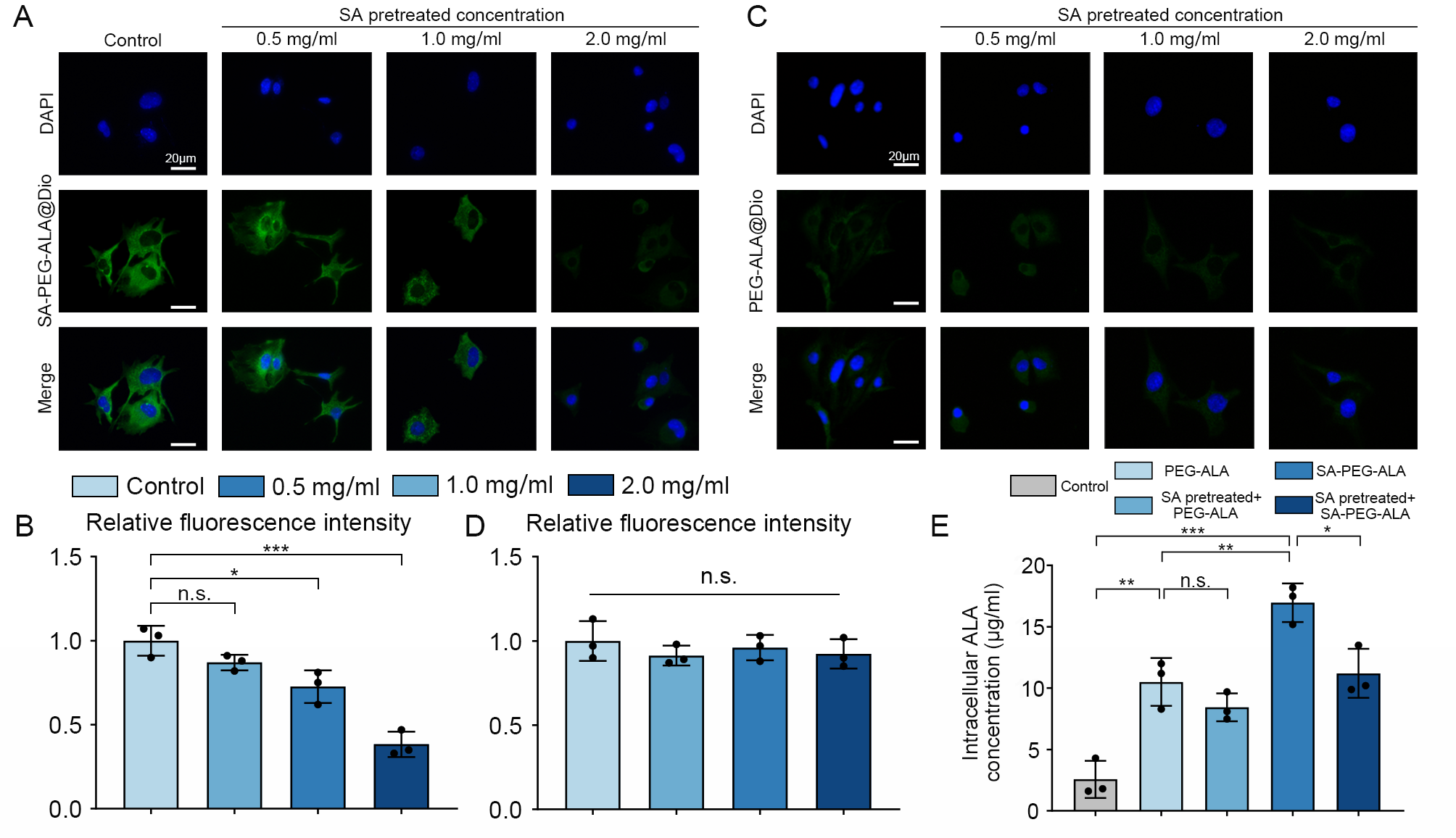

Supplement: Supplementary 1 — Figs. S1 to S9 Tables S1 to S7 [file bmr.0305.f1.zip › supplymentary_figure5.tif]

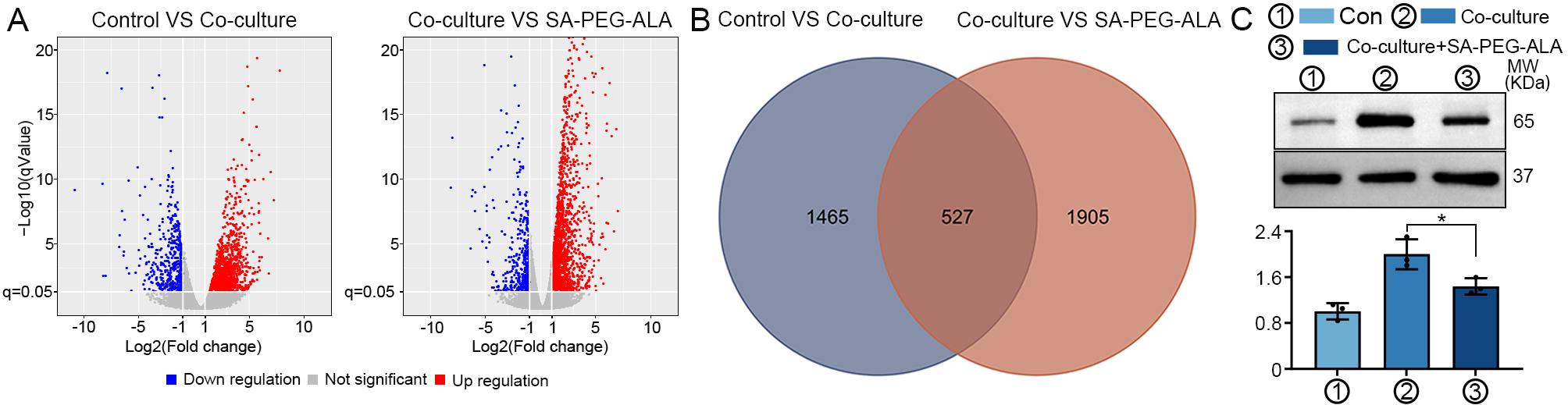

Supplement: Supplementary 1 — Figs. S1 to S9 Tables S1 to S7 [file bmr.0305.f1.zip › supplymentary_figure8.tif]

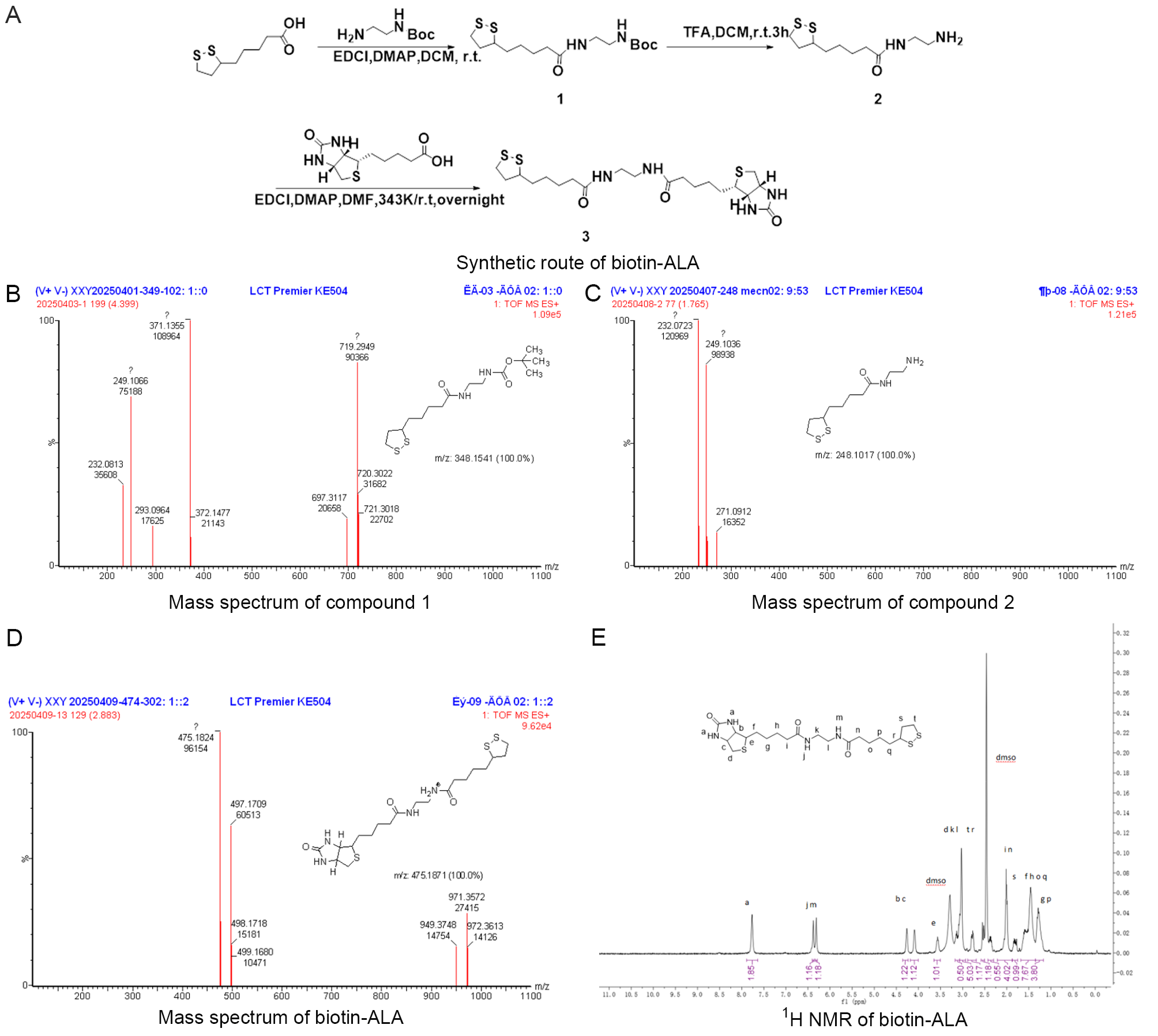

Supplement: Supplementary 1 — Figs. S1 to S9 Tables S1 to S7 [file bmr.0305.f1.zip › supplymentary_figure9.tif]
