# Supplementary material for: E-Selectin-Targeted Nanomicelles via Sialic Acid Conjugation for Anti-Inflammatory Efficacy and Alleviating the Progression of Metabolic-Associated Steatotic Liver Disease
Source: Biomater Res. 2026 Feb 2;30:0305. doi: 10.34133/bmr.0305 (PMC12862626; doi:10.34133/bmr.0305)
Supplement: Supplementary 1 — Figs. S1 to S9 Tables S1 to S7 [file bmr.0305.f1.zip › Supporting data_revised.doc]

**Supporting data**

1. **Supplementary Tables**

Table S1. List of previously reported endothelial cells “candidate genes” involved in leukocyte adhesion

| Gene Name | Coding Protein | log_2_FC | Pvalue | Qvalue |
| --- | --- | --- | --- | --- |
| *SELE* | E-selectin | 2.11343 | 0.00019 | 0.00342 |
| *ICAM1* | Icam-1 | 1.78030 | 0.00504 | 0.03965 |
| *VCAM1* | Vcam-1 | 1.18260 | 0.07229 | 0.24953 |
| *CD34* | CD34 | 1.05710 | 0.18160 | 0.43935 |
| *ICAM-2* | ICAM-2 | 0.56076 | 0.81522 | 0.94825 |
| *SCD1* | SCD1 | 0.45551 | 0.60711 | 0.81722 |
| *JAM2* | JAM2 | -0.3099 | 0.15812 | 0.40250 |
| *JAM3* | JAM3 | -0.3381 | 0.09021 | 0.28663 |
| *SELP* | P-selectin | -0.8758 | 0.05627 | 0.21307 |

Table S2. Detailed docking results of SA molecules and receptor SELE

| No. | Hydrogen bond interaction | Hydrogen bond distance |
| --- | --- | --- |
| ligand | Tyr214、Trp265、Asn266、Thr268 | 1.98、2.76、2.86、3.70 |

Table S3. Detailed interaction forces between SA and SELE

| Energy (Kcal/mol) | Item | Value |
| --- | --- | --- |
| Estimated Free Energy of Binding | Combined free energy | -3.31 |
| Estimated dissocation Constant , Kd | Dissociation constant | 3.74 |
| (1) Final Intermolecular Energy | Intermolecular interaction | -9.28 |
| VDW+Hbond+desolv Energy | Van der Waals force + hydrogen bond + desolvation | -9.28 |
| Electrostatic energy | Electrostatic potential energy | 0.0 |
| (2) Final Total Internal Energy | Internal molecular energy | -14.28 |
| (3) Torsional Free Energy | Torsional free energy | 5.97 |
| (4) Unbound System’s Energy | Non-bonded system energy | -14.28 |

The calculation formula for the binding free energy is: [(1)+(2)+(3)-(4)]. The units of all energy values are kcal/mol. The conversion formula for the Kd value is: Kd = In (△G/RT), where R is the constant 8.314 J/mol·K and T is the temperature 298.15 K. The smaller the Kd value, the stronger the binding.

Table S4. Kinetic and affinity parameters.

| [Parameter](../../../../F:/微信文件/WeChat%20Files/wxid_bxvu8kneipwr22/FileStorage/File/2024-10/javascript:%3B) | [Paraphrase](../../../../F:/微信文件/WeChat%20Files/wxid_bxvu8kneipwr22/FileStorage/File/2024-10/javascript:%3B) | Result |
| --- | --- | --- |
| ka (1/(M*s)) | Association rate constant | 7.16e+02 |
| kd (1/s) | Dissociation rate constant | 3.71e-05 |
| KD (M) | The dissociation equilibrium constant, also called the affinity constant | 4.16e-06 |

Table S5. Patients’ characteristics in each category

|  | Age  (years) | Gender (M/F) | BMI (kg/m^2^) | AST  (U/L) | ALT  (U/L) |
| --- | --- | --- | --- | --- | --- |
| Normal  liver | 49 | F | 31.5 | 25 | 27 |
|  | 58 | M | 34.0 | 33 | 25 |
|  | 63 | M | 32.4 | 41 | 24 |
| MASH  F0-1 | 42 | M | 38.2 | 69 | 42 |
|  | 60 | F | 31.1 | 60 | 39 |
|  | 29 | M | 37.8 | 86 | 55 |

Gender (M/F) M:male; F:female

Table S6. Primary antibodies for WB, IHC and IF

| Antibody | WB | IHC | IF | Specificity | Catalog |
| --- | --- | --- | --- | --- | --- |
| SELE | 1:1000 | 1:100 | 1:100 | Rabbit monoclonal | ab322206 |
| SELE |  |  | 1:100 | Alexa Fluor^®^ 488 Rabbit monoclonal | [ab318169](https://www.abcam.cn/products/primary-antibodies/alexa-fluor-488-cd62e-antibody-epr25616-62-ab318169.html) |
| ICAM-1 | 1:1000 |  |  | Rabbit monoclonal | ab179707 |
| CD68 |  | 1:100 |  | Rabbit polyclonal | Ab303565 |
| NFκB | 1:1000 |  | 1:100 | Rabbit monoclonal | 8242S |
| p-NFκB | 1:1000 |  |  | Rabbit monoclonal | 3033S |
| ACTA2 | 1:1000 |  |  | Mouse monoclonal | ab7817 |
| BAX | 1:1000 |  |  | Rabbit monoclonal | ab32503 |
| BCL2 | 1:1000 |  |  | Rabbit monoclonal | ab182858 |
| αSMA |  | 1:100 |  | Rabbit monoclonal | 19245T |
| p-IKB alpha | 1:1000 |  |  | Rabbit monoclonal | 2859S |
| IKB alpha | 1:1000 |  |  | Rabbit monoclonal | ab32518 |
| IKK-alpha | 1:1000 |  |  | Rabbit monoclonal | ab32041 |
| IKK-beta | 1:1000 |  |  | Rabbit monoclonal | ab124957 |
| HSP70 | 1:1000 |  |  | Mouse monoclonal | ab2787 |
| Anti-Myc tag | 1:1000 |  |  | Rabbit monoclonal | ab9106 |
| Anti-HA tag | 1:1000 |  |  | Mouse polyclonal | ab1424 |
| GAPDH | 1:1000 |  |  | Rabbit monoclonal | ab9485 |

Table S7. Sequence of Primers for PCR

| Primers |  | Sequences (5’------3’) |
| --- | --- | --- |
| *SELE* | Forward： | 5’-CCAATCTGAAACATTCACCGAGT-3’ |
|  | Reverse： | 5’-GAGTCTTTGGTTCGTTGGATGTA-3’ |
| *SCD1* | Forward： | 5’-TTCTTGCGATACACTCTGGTGC-3’ |
|  | Reverse： | 5’-CGGGATTGAATGTTCTTGTCGT-3’ |
| *CD36* | Forward： | 5’-CCTGCAAATGTCAGAGGAAA-3’ |
|  | Reverse： | 5’-GCGACATGATTAATGGCACA-3’ |
| *ACTA2* | Forward： | 5’-TCCTGACGCTGAAGTATCCGATA-3’ |
|  | Reverse： | 5’GGTGCCAGATCTTTTCCATGTC--3’ |
| *COL1A1* | Forward： | 5’-GCTTGAAGACCTATGTGGGTATAA-3’ |
|  | Reverse： | 5’-GGGTGGAGAAAGGAACAGAAA-3’ |

2. **Supplementary Figures**

Figure S1. SELE was overexpressed in murine MASH. (A) The expression of *SELE* and *ICAM-1* from GEO dataset (GSE83452). (B) H&E staining was used to analyze HFHC-induced MASH model, scale bar = 100 μm. (C) Hepatic relative *SELE* exprssion in MASH or normal mice. (D) The correlation of *SELE* exprssion and NAS. Data are means ± SD of three independent experiments.

Figure S2. Mass spectrum of SA-PEG-ALA. (A) Mass spectrum of SA-PEG-ALA. (B, C) The important intermediates from synthesis pathway.

Figure S3. Design and synthesis of PEG-ALA nanomicelles. (A) Synthetic route of PEG-ALA. (B) Mass spectrum of PEG-ALA.

Figure S4. Design and synthesis of SA-PEG. (A) Synthetic route of SA-PEG. (B) Mass spectrum of compound 1. (C) Mass spectrum of SA-PEG. (D) ^1^H NMR of SA-PEG. (E) The release of ALA from free ALA, PEG-ALA or SA-PEG-ALA in PBS at pH7.4.

Figure S5. SA treatment inhibited SA-PEG-ALA internalized by LSECs. (A, B) Immunofluorescent staining and quantification of SA-PEG-ALA internalization by LSECs in indicated group, scale bar = 20 μm. (C, D) Immunofluorescent staining and quantification of PEG-ALA internalization by LSECs in indicated group, scale bar = 20 μm. (E) HPLC was used to detect the intracellular ALA concentration in LSECs in indicated group, n = 3 per group. Statistical significance was determined via a one-way ANOVA with Tukey test. Results are means ± SD of three independent experiments. ^*^*P*<0.05, ^**^*P*<0.01, ^***^*P*<0.001.

Figure S6. SA-PEG-ALA attenuated steatosis and inflammation in an HFHC-induced MASH model. (A) The gross image of mice in the indicative groups, scale bar = 1 cm. (B) The curve of body weight in the indicative groups. (C) The body weight and liver weight in the indicative groups. (D) The liver/body weight ratio in the indicative groups. (E, F) Representative microscopic images and quantitative analysis of Oil red O staining in the indicative groups, scale bar = 50 μm, n = 8 mice per group. (G) Representative images of H&E staining of liver sections from indicative groups, n = 8 mice per group. Arrows indicate immune cells infiltrates. Scale bar = 50 μm. Data are means ± SD of three independent experiments. ^*^*P*<0.05, ^***^*P*<0.001. n.s., not significant.

Figure S7. Safety evaluation of different treatments on HFHC-induced MASH model. (A) H&E histopathological sections of heart, lung, kidney and spleen excised from normal chow fed healthy mice or HFHC-fed mice three days after the final injection of various formulations, scale bar=80μm, n = 8 mice per group. (B) Organ coefficient in indicative groups, n = 8 mice per group. (C) The renal function parameters of BUN and CRE in normal chow-fed healthy mice or HFHC-fed mice three days after the final injection of various formulations, n = 8 mice per group. (D) Different concentrations of different drugs on cell viability. Data are means ± SD of three independent experiments. n.s., not significant.

Figure S8. SA-PEG-ALA inhibited steatotic hepatocytes induced endothelial dysfunction. (A) Volcano plot representing regulated genes with fold change. (B) Venn diagram representing the screened genes in the intersection part with A. (C) Western blots and quantitative analysis of SELE in LSECs in the indicated groups. Data are means ± SD of three independent experiments. ^*^*P*<0.05.

Figure S9. Design and synthesis of biotin-ALA. (A) Synthetic route of biotin-ALA. (B) Mass spectrum of compound 1. (C) Mass spectrum of compound 2. (D) Mass spectrum of biotin-ALA. (E) ^1^H NMR of biotin-ALA.

**Full length WB for three repetition**

Figure2 full length WB

Figure3 full length WB

Figure7 full length WB

Figure8 full length WB

Figure9 full length WB

Figure10 full length WB
